# Melanoma cell-derived exosomes in plasma of melanoma patients suppress functions of immune effector cells

**DOI:** 10.1038/s41598-019-56542-4

**Published:** 2020-01-09

**Authors:** Priyanka Sharma, Brenda Diergaarde, Soldano Ferrone, John M. Kirkwood, Theresa L. Whiteside

**Affiliations:** 1 Department of Pathology, University of Pittsburgh School of Medicine and UPMC Hillman Cancer Center, Pittsburgh, PA 15213 USA; 2 Department of Human Genetics, Graduate School of Public Health, University of Pittsburgh and UPMC Hillman Cancer Center, Pittsburgh, PA 15213 USA; 3 Department of Surgery, Massachusetts General Hospital and Harvard Medical School, Boston, MA 02114 USA; 4 Department of Medicine, University of Pittsburgh School of Medicine and UPMC Hillman Cancer, Pittsburgh, PA 15213 USA; 5 Departments of Immunology and Otolaryngology, University of Pittsburgh School of Medicine, Pittsburgh, PA 15213 USA

**Keywords:** Biomarkers, Cancer

## Abstract

Melanoma patients’ plasma contains exosomes produced by malignant and normal cells. Plasma exosomes were isolated and separated by immunocapture into two fractions: melanoma cell-derived exosomes (MTEX) and normal cell-derived exosomes (non-MTEX). Immunosuppressive effects of MTEX on primary human immune cells were evaluated. Exosomes were isolated from plasma of 12 melanoma patients and six healthy donors (HDs). Expression levels of 19 immunoregulatory proteins in MTEX, non-MTEX and HDs exosomes were evaluated by on-bead flow cytometry. Functional/phenotypic changes induced in CD8^+^ T or natural killer (NK) cells by MTEX or non-MTEX were compared. Plasma protein levels were higher in patients than HDs (*P* < 0.0009). In patients, MTEX accounted for 23–66% of total exosomes. MTEX were enriched in immunosuppressive proteins (*P* = 0.03). MTEX, but not HDs exosomes, inhibited CD69 expression (*P* ≤ 0.0008), induced apoptosis (*P* ≤ 0.0009) and suppressed proliferation (*P* ≤ 0.002) in CD8^+^ T cells and downregulated NKG2D expression in NK cells (*P* = 0.001). Non-MTEX were enriched in immunostimulatory proteins (*P* = 0.002) and were only weakly immunosuppressive. Elevated MTEX/total exosome ratios and, surprisingly, non-MTEX ability to induce apoptosis of CD8^+^ T cells emerged as positive correlates of disease stage. MTEX emerge as the major mechanism of tumor-induced immune suppression and as an underestimated barrier to successful melanoma immunotherapy.

## Introduction

Melanoma is among the most aggressive human cancers and its incidence is increasing worldwide^1^. Immune suppression is a common manifestation of disease progression in melanoma thought to contribute to tumor escape from the host immune system^2^. Mechanisms responsible for tumor-induced immune suppression in melanoma include PD-L1 overexpression in the tumor, and PDL-L1+ tumor-derived exosomes are reported to interfere with patients’ responses to oncological immunotherapies^3,4^. Recent gains achieved with checkpoint inhibitory therapies in melanoma suggest that rejuvenation of anti-tumor immune activity improves disease outcome^5,6^. However, many melanoma patients remain unresponsive to checkpoint inhibition for reasons that are not clear^7^. Circulating melanoma cell-derived exosomes (MTEX) enriched in suppressive proteins might represent such an immunoinhibitory mechanism^8,9^.

Exosomes are a part of the tumor-immune cell communication system^10^. Tumors produce and release into body fluids numerous extracellular vesicles (EVs), including a small (30–150 nm sized) subset of vesicles derived from the endocytic compartment of tumor cells^11^. Melanoma cells produce more exosomes than normal melanocytes, and melanoma patients’ plasma contains increased levels of exosomes carrying melanoma-associated antigens (MAAs), immunosuppressive proteins (FasL, TGF-β) and oncoproteins, including Myc^8,9,12,13^. Exosomes isolated from supernatants of melanoma cell lines inhibit functions of primary human immune cells *in vitro*^8,14^. Exosomes in melanoma patients’ plasma are immunosuppressive *in vivo*^3^. Nevertheless, the key role exosomes play in tumor escape from the host immune system remains unconfirmed and may be underestimated.

Exosomes represent a heterogenous population of vesicles produced by all cells, carry diverse molecular and genetic cargos and circulate freely through body fluids^15,16^. Cancer patients’ plasma contains exosomes produced by malignant and normal cells. We recently reported a method for immune capture of melanoma-cell derived exosomes (MTEX) from patients’ plasma^17^, and have successfully separated MTEX from normal cell-derived exosomes (non-MTEX)^17–20^. Here, we show that the ratios of MTEX/non-MTEX in plasma differ among patients with advanced metastatic disease. While MTEX carry an abundance of immunosuppressive proteins and inhibit functions of human primary immune cells *ex vivo*, non-MTEX stimulate immune cell activity. The phenotypic and functional characterization of MTEX emphasizes their potential role as biomarkers of melanoma progression.

## Methods

### Study participants and biospecimens

The study population consists of 12 melanoma patients and 6 healthy donors (HDs) used as controls. Selected characteristics of all subjects are listed in STable 1. Age at blood draw did not differ between patients and healthy controls (*P* = 0.78); 5 patients were NED and 7 has metastatic disease. Patients were seen at the UPMC Hillman Cancer Center; data and samples were obtained from the University of Pittsburgh Melanoma SPORE Bank (IRB #970186). For HDs, data and samples were collected via IRB-approved protocol #991206. All study participants provided written informed consent. Blood samples were processed; plasma was separated, aliquoted into 2 mL cryotubes and stored at −80 °C until thawed for exosome isolation.

### Antibodies and reagents

The mAbs 225.28 and 763.74, which recognize distinct and spatially distant epitopes of CSPG4, and MICA-specific mAbs WW98B and WJ-1 were developed and characterized as described^18–20^. These mAbs were purified from ascitic fluid by affinity chromatography on a Protein G column, and their purity was monitored by SDS-PAGE. Reactivity of mAb preparations was tested in binding assays with cells expressing the target antigens. The epitopes recognized by mAbs 225.28 and 763.74 are selectively present on melanoma cells^20^ and are expressed by most, if not all, melanoma cells in >85% of human melanomas. Both epitopes are carried on MTEX^17^. One of these mAbs was used for immune capture on beads and the other for antigen detection by on-bead flow cytometry^17^.

Additional antibodies used were purchased from commercial vendors and are listed in Supplementary Methods.

### Isolation of exosomes from plasma and determination of protein content

Thawed plasma samples were pre-cleared by centrifugation and ultrafiltration prior to mini Size Exclusion Chromatography (miniSEC) as described^21^. Exosomes were eluted in the void volume with PBS and harvested in fraction #4. Protein content of fraction #4 was determined using the BCA protein assay kit [Pierce Biotechnology, Rockford, IL, USA]; vesicle size and particle numbers were verified using qNano (Izon) as described^21,22^.

### Immunocapture of MTEX from plasma exosomes and recovery of non-MTEX

For immunocapture of MTEX from fraction #4 exosomes, CSPG4 mAbs (clone 763.64 or 225.28) were biotinylated using a one-step antibody biotinylation kit (Novus Biologicals) as per the manufacturer’s protocol. Exosomes (10 µg protein) were immunocaptured on biotin-labeled mAb-charged streptavidin magnetic beads as described^17^ The non-captured fraction was re-captured on beads charged with biotinylated anti-CD63 mAb for antigen detection^17^. Protein levels of MTEX (immunocaptured exosomes) were calculated by subtracting protein levels in the non-captured fraction (non-MTEX) from protein levels in total exosomes.

### Exosome protein detection by on-beads flow cytometry

On-bead flow cytometry analysis of MTEX and non-MTEX was performed using a modified method reported by Kastresana and Jones^23^ as described^24^.

Flow cytometry on-beads exosome profiling was performed using Gallios TM flow cytometer (Beckman Coulter). Results were expressed in Relative Fluorescence Intensity (RFI) values, where RFI = MFI (mean fluorescence intensity) of detection mAb/MFI of isotype control.

To confirm that flow-cytometry based antigen detection (RFIs) in exosomes is equivalent to Western blots (WBs), expression of several inhibitory proteins on MTEX and non-MTEX obtained from 2 melanoma patients (#8 and #9) was evaluated by both detection methods (SFig. 1d).

### Functional studies with MTEX and non-MTEX

Primary activated T or NK cells of each patient were co-incubated with aliquots of MTEX, non-MTEX or total exosomes from fraction #4 to compare inhibitory effects of exosomes on immune cell functions. MTEX captured on beads were used in these assays, while total exosomes and non-MTEX were not captured on beads. The presence of beads alone or beads coated with biotinylated anti-CSPG4 mAbs did not interfere with functional assays (see SFig. 6a; the data are for MTEX-induced apoptosis). Ab-coated beads alone were found to be equivalent to no-exosome controls in other functional assays as well (data not shown).

The following functional assays were performed following co-incubation of CD8^+^ T cells with exosomes (See Supplementary Methods for details).

- CD69 downregulation on T cells
- Changes in CD69 mRNA transcripts
- NF-κB activation in CD8^+^ T cells
- Apoptosis of CD8^+^ T cells
- CFSE-based proliferation assays
- NKG2D down-regulation on NK cells.

### Statistical analyses

Flow cytometry analyses were performed with Kaluza version 1.5 (Beckman Coulter). Wilcoxon signed-rank tests were used to evaluate differences between paired samples; Wilcoxon-Mann-Whitney tests were used to evaluate differences between different subject groups. An MAA RFI score was created for each subject by summing their RFI values for CSPG4, TYRP2, MelanA, Gp100, and VLA4. A similar approach was used to create an immunosuppressive RFI score (includes: PDL-1, CD39, CD73, FasL, LAP-TGFβ, TRAIL, and CTLA-4) and an immunostimulatory RFI score (includes: CD40, CD40L, CD80, OX40, and OX40L) for each subject. A Stimulatory/Suppressive (stim/supp) ratio was calculated for each subject by dividing their immunostimulatory RFI score by their immunosuppressive RFI score. The different proteins were also evaluated individually. Spearman’s correlation coefficients (r) were calculated to examine whether variables correlated with each other. *P* values < 0.05 were considered significant. Statistical analyses were performed using the SAS® statistical software package (SAS version 9.4, SAS Institute Inc., Cary, NC, USA) and GraphPad Prism (version 7.03, GraphPad Software Inc., La Jolla, CA, USA).

### Ethics approval and consent to participate

Blood samples from melanoma patients and healthy subjects were collected under University of Pittsburgh Institutional Review Board approved protocols #970186 and #991206, respectively. All subjects provided written informed consent. All experiments were performed in accordance with relevant guidelines and regulations.

## Results

### MTEX and non-MTEX recovery after immunocapture

Immunocapture of MTEX was performed using total exosomes isolated from plasma of patients or HDs by miniSEC as we previously reported^21^. Briefly, this procedure is a 3-step process: (a) isolation of “total exosomes”, from plasma by SEC and their recovery as fraction #4; (b) separation of total exosomes into MTEX and non-MTEX using streptavidin beads coated with biotinylated anti-CSPG4 mAbs; and (c) recovery of MTEX on beads and capture of non-MTEX on beads coated with anti-CD63 mAbs. Protein levels in total exosomes (fraction #4) to be immunocaptured were normalized to 1 mL of every individual’s plasma used for miniSEC. Exosomes isolated from patients and HDs had similar morphology and size (SFig. 1a,b). The number of exosomes isolated from patients ranged from 1.64 × 10^11^/mL to 2.68 × 10^11^/mL; for HDs from 3.22 × 10^10^/mL to 8.6 × 10^10^/mL (SFig. 1b). WBs of exosomes from patients or HDs confirmed their endocytic origin; they all contained TSG101 protein (SFig. 1c,d). Specificity of the immunocapture for melanoma exosomes was verified by showing that: (i) consistently, non-MTEX were CSPG4(−); only MTEX were CSPG4(+) (SFig. 2a,b); (ii) exosomes from HDs’ plasma were negative for CSPG4 (SFig. 2c); (iii) only MTEX were highly enriched in MAAs (SFig. 4a); (iv) MTEX were CSPG4 (+) but CD3(−); only non-MTEX carried CD3 (SFig. 2d); (v) in spiking experiments, where melanoma exosomes were added to exosomes obtained from HDs (1:1), the captured fraction contained all CSPG4(+) exosomes, while the non-captured fraction was CSPG4(−) (data not shown).

Total exosome protein levels were higher in patients than in HDs (mean 76 µg/mL versus 54 µg/mL; *P* = 0.0009; SFig. 3a) and were not significantly different between melanoma patients with no evident disease (NED) or active metastatic disease at blood draw or between male and female patients (SFig. 3b). MTEX protein levels were also not significantly different between these sub-groups (SFig. 3c). Among the 12 melanoma patients, MTEX represented 23% to 66% of total plasma exosomes (mean: 44%). High percentages of MTEX among plasma exosomes of patients with metastatic melanoma are not unexpected, although the reasons for elevated MTEX in 5/12 patients who are clinically NED in our cohort are not clear and without further evidence for disease recurrence cannot be attributed to a micrometastatic disease. Pre-clearing of plasma prior to miniSEC removes EVs larger than 150 nm, which presumably represent “contaminating” platelet-derived or RBC-derived vesicles. Also, non-MTEX were largely CD3^+^ (SFig. 2d), indicating their origin from T cells and not from platelets or RBCs. No significant differences were observed in the MTEX/total exosome protein ratio between NED patients and those with active metastatic disease or between male and female patients (SFig. 3c). However, the MTEX/total exosome protein ratios for melanoma patients fell into two categories: low (around 0.3) and high (0.5 to 0.7). This finding suggests that the MTEX/total exosome protein ratios could potentially discriminate between patient cohorts with distinct clinical presentations and could be an important endpoint for further investigations.

### Protein profiles of MTEX and non-MTEX

Proteins present in MTEX and non-MTEX were initially studied by WBs, and before moving on to on-bead flow cytometry, MTEX and non-MTEX protein cargos were evaluated using both methods. SFigure 1d shows that WB profiles of MTEX and non-MTEX for several immunosuppressive proteins are comparable with RFI values for patients #8 and #9. Flow cytometry is advantageous for detection of proteins on the exosome surface because it is quantitative, highly reproducible (see Methods), and requires less protein than WBs.

**Figure 1:**
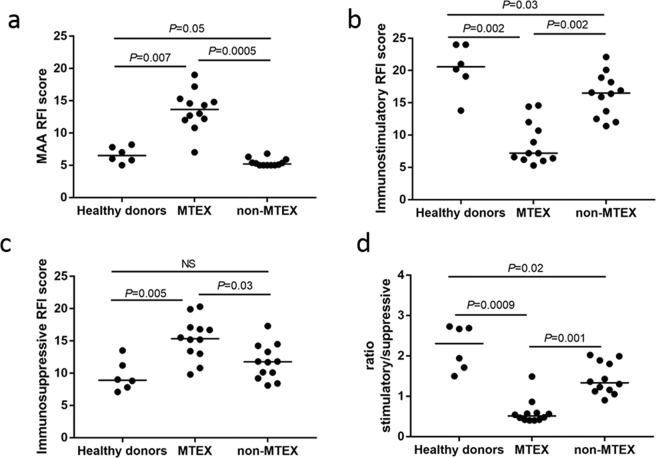
The RFI scores for: (**a**) MAAs, (**b**) immunostimulatory proteins and (**c**) immunosuppressive proteins carried by total exosomes from plasma of HDs, and by MTEX and non-MTEX from plasma of melanoma patients. In (**d**) the stimulatory/suppressive (stim/supp) ratio for HDs exosomes and for MTEX and non-MTEX are shown. The MAA RFI score includes CSPG4, TYRP2, MelanA, Gp100, and VLA4; the immunostimulatory RFI score includes CD40, CD40L, CD80, OX40, and OX40L; the immunosuppressive RFI score includes PDL-1, CD39, CD73, FasL, LAP-TGFβ, TRAIL, and CTLA-4. Wilcoxon signed-rank tests were used to evaluate differences between MTEX and non-MTEX; Wilcoxon-Mann-Whitney tests were used to evaluate differences between patients and healthy controls. Horizontal bars indicate median values. NS: no significant difference.

Representative flow cytometry data for MTEX and non-MTEX of one patient (#8) for all evaluated surface proteins are shown in SFig. 4. For each subject, STable 2 lists the RFI values for individual MAA or immunoregulatory proteins (n = 19) carried by MTEX, non-MTEX or HD-exosomes as well as the created scores. The MAA RFI s was significantly higher for MTEX than for non-MTEX or HDs exosomes (Fig. 1a and STable 2). While the same immunoregulatory proteins were detectable in MTEX, non-MTEX and HDs exosomes, *quantitative* differences readily discriminated between these exosome subsets (STable 2). The immunostimulatory RFI score was significantly lower for MTEX than for non-MTEX or HDs exosomes (Fig. 1b). The immunosuppressive RFI score was significantly higher for MTEX than for non-MTEX; the score for non-MTEX was similar to that for HDs exosomes (Fig. 1c). The stimulatory/suppressive (stim/supp) ratio for MTEX was significantly lower than the ratio for non-MTEX and HDs exosomes (mean, respectively, 0.6, 1.4 and 2.2; Fig. 1d).

**Figure 2:**
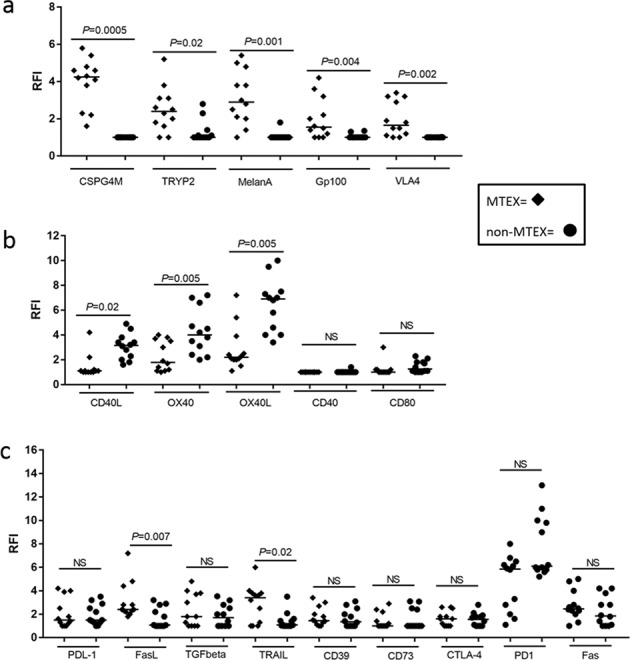
The RFI values for: (**a**) individual MAAs, (**b**) individual immunostimulatory proteins and (**c**) individual immunosuppressive proteins as determined by on-bead flow cytometry for all 12 melanoma patients. Horizontal bars indicate median values. NS: no significant difference. Wilcoxon signed-rank tests were used to evaluate differences between MTEX and non-MTEX.

The different proteins in exosome cargos were also evaluated individually (Fig. 2). Significant differences in RFI scores between MTEX and non-MTEX were observed for all MAA proteins, which were largely absent in non-MTEX or HDs exosomes (STable 2). Among the immunosuppressive proteins, FasL (*P* = 0.007) and TRAIL (*P* = 0.02) were highly enriched in the MTEX fractions (Fig. 2c). In contrast, CD40L (*P* = 0.02) as well as OX40 and OX40L (both *P* = 0.005) were highly enriched in the non-MTEX fractions (Fig. 2b). Interestingly, most non-MTEX also carried substantial levels of several immunosuppressive proteins (Fig. 2c). The mean RFIs for PD-L1 on MTEX and non-MTEX were 1.6 and 1.9, respectively, and in 5/12 patients, PD-L1 was similarly enriched in both MTEX and non-MTEX.

Spearman correlation coefficients were calculated to explore correlations among the proteins carried by MTEX and non-MTEX (SFig. 5). Interestingly, significant correlations were more common in non-MTEX than MTEX, suggesting that the protein cargos of MTEX may be more diverse than those in non-MTEX.

### Immunosuppressive functions of MTEX versus non-MTEX

Immunoregulatory functions of MTEX, non-MTEX and HDs exosomes were evaluated in co-incubation experiments with human primary immune cells (CD8^+^ T cells or NK cells).

#### Inhibition of T-cell activation upon co-incubation with MTEX

**Figure 3:**
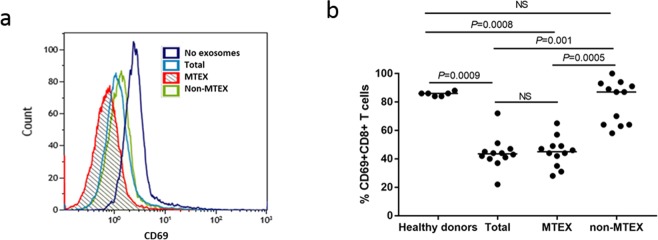
Exosome-mediated effects on CD69 expression levels in CD8^+^ T cells. MTEX were immunocaptured from patients’ plasma as described in Methods and were co-incubated with primary human activated CD8^+^ T cells for 6 h at 37 °C. CD69 expression levels on T cells were measured by flow cytometry. (**a**) Representative exosome-mediated downregulation of CD69 expression levels on activated CD8^+^ T cells after co-incubation with exosomes as indicated. (**b**) % of CD69 + CD8^+^ T cells after co-incubation with HDs’ exosomes or total exosomes, MTEX or non-MTEX obtained from melanoma patients’ plasma. Horizontal bars indicate median values. All data are normalized to no exosome (PBS) control for each patient/HD individually. Wilcoxon signed-rank tests were used to evaluate differences between paired samples (e.g., between MTEX and non-MTEX); Wilcoxon-Mann-Whitney tests were used to evaluate differences between patients and HDs. NS: no significant difference.

When activated CD8^+^ T cells were co-incubated with MTEX or total exosomes, levels of CD69 protein on the surface of CD8^+^ T cells and the % of CD69^+^CD8^+^ T cells were significantly decreased (*P* = 0.0009). No CD69 downregulation was seen after co-incubation with HDs exosomes or non-MTEX (Fig. 3). Notably, in 5/12 melanoma patients, non-MTEX also mediated CD69 downregulation on T cells.

Our previous studies showed that after 6 h co-incubation with human primary T cells, tumor-derived exosomes (TEX) were not internalized but remained at the T-cell surface^25^. This suggests that CD69 downregulation on T cells after 6 h co-incubation with MTEX may be a cell surface signaling event. Using qRT-PCR, we demonstrated that total exosomes and MTEX, but not non-MTEX, induced down-regulation of CD69 mRNA transcripts in CD8^+^ T cells (SFig. 6a). Further, a 30 min co-incubation of CD8^+^ T cells with MTEX induced translocation of the NF-κB subunit p65 to the nucleus of recipient T cells, confirming activation of the NF-κB pathway in T cells responding to MTEX-mediated signaling (SFig. 6b). The identity of suppressive signals that induced CD69 down-regulation is unknown. We previously reported reduced CD69 expression levels on primary CD8^+^ T cells by PD-L1^high^ exosomes from plasma of patients with head and neck cancer^24^. Blocking with anti-PD-1 Abs partly restored CD69 expression levels in the recipient T cells, suggesting that exosomes carrying PD-L1 were involved^24^. In the current study, downregulation of CD69 expression on activated CD8^+^ by MTEX, non-MTEX or total exosomes was partially blocked by anti-PD-1 mAbs (SFig. 7). CD69 expression was in part restored (~30%) by CD8^+^ T cells after the anti-PD1 mAb block, but this amelioration of CD69 expression did not correlate with levels of PD-L1 protein carried by MTEX in melanoma patients (*r* = −0.01, *P* = 0.98) or with expression levels of any of the other immunoregulatory proteins we evaluated (data not shown).

#### Suppression of CD8+ T-cell proliferation by MTEX

**Figure 4:**
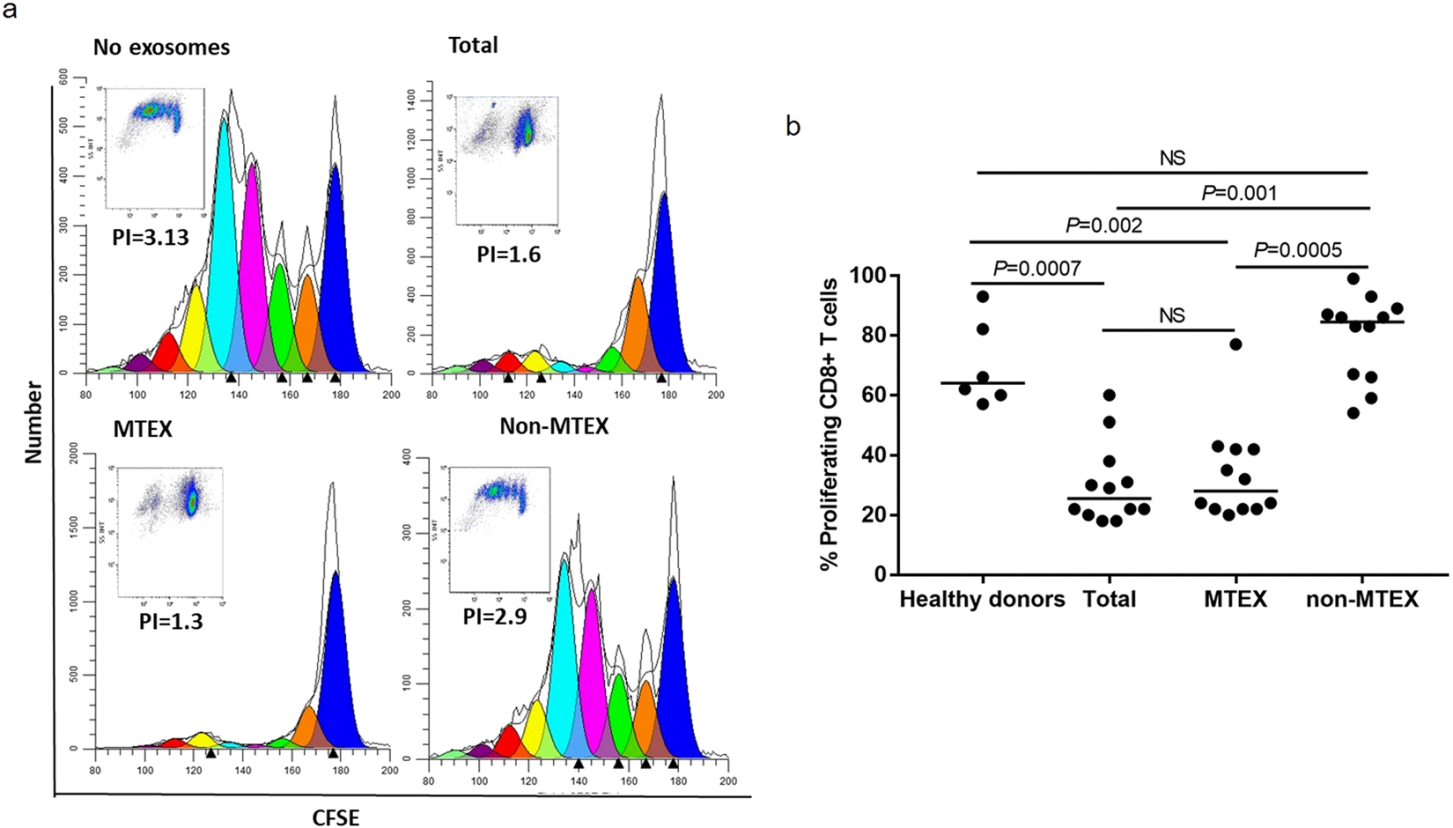
Exosome-induced suppression of CD8^+^ T cell proliferation. (**a**) Representative CSFE-assay for exosomes from one patient showing proliferation inhibition induced in CD8^+^ T cells by total exosomes, MTEX and non-MTEX. No suppression was seen with non-MTEX. (**b**) CD8^+^ T cell proliferation after co-incubation with HDs exosomes or total exosomes, MTEX or non-MTEX obtained from melanoma patients’ plasma. Note that non-MTEX in 4/12 patients mediated suppression. Data were normalized to no exosome (PBS) control. Horizontal bars indicate median values. NS: no significant difference. Wilcoxon signed-rank tests were used to evaluate differences between paired samples; Wilcoxon-Mann-Whitney tests were used to evaluate differences between patients and HDs. Results for blocking of MTEX-induced suppression of CD8^+^ T cell proliferation are presented in SFig. 8.

The exosome fractions isolated from patients’ and HDs’ plasma were also evaluated for their ability to suppress proliferation of activated CD8^+^ T cells. Figure 4a shows that suppression of CD8^+s^ T-cell proliferation is mediated by MTEX and total exosomes but not by non-MTEX (representative data). MTEX-mediated suppression of proliferation was comparable to that induced by total exosomes (Fig. 4b). Compared to MTEX, non-MTEX and HDs exosomes were minimally suppressive in proliferation assays *P* = 0.0005). Surprisingly, MTEX-mediated suppression of CD8^+^ T cell proliferation did not correlate with the expression level of any single immunoregulatory protein included in the profiling panel (Fig. 2, but rather reflected the sum of all inhibitory signals. Importantly, non-MTEX-mediated suppression of proliferation was seen in only 4/12 patients (Fig. 4b), it correlated with TRAIL levels carried by these exosomes (*r* = 0.60. *P* = 0.04).

To determine whether MTEX-mediated suppression of T-cell proliferation involved surface protein interactions, we used neutralizing Abs or pharmacological inhibitors of the inhibitory proteins carried by MTEX to block immune suppression (SFig. 8a). MTEX-induced suppression of CD8^+^ T-cell proliferation was blocked by anti-PD1 mAb, anti-TGF-β mAb, the NF-κB inhibitor, and by heat denaturation of MTEX. The CD73 inhibitor or anti-Fas mAb failed to restore T-cell proliferation.

#### MTEX-mediated apoptosis in CD8^+^ T cells

**Figure 5:**
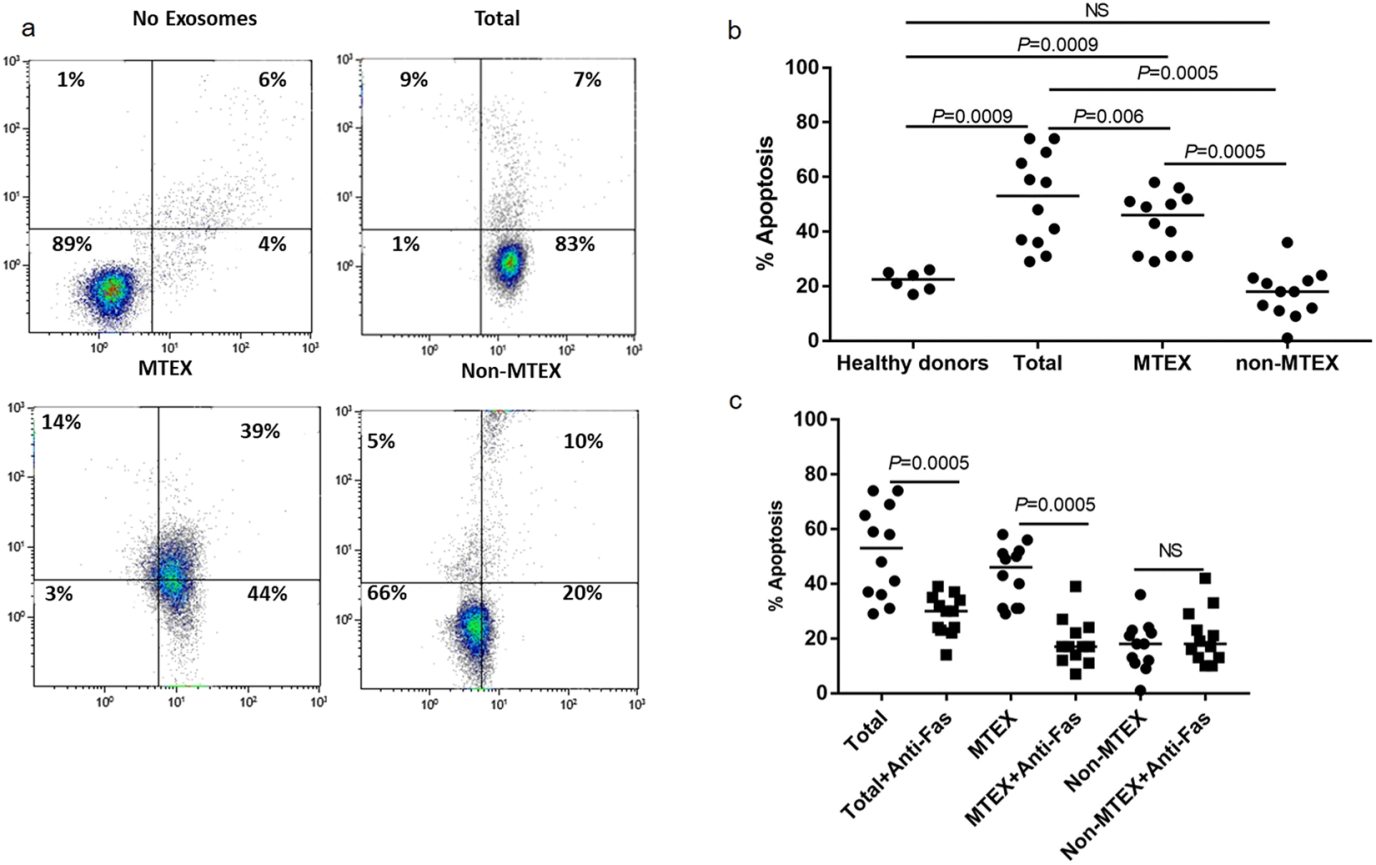
Exosome-mediated apoptosis in primary activated CD8^+^ T cells. (**a**) Representative flow cytometry data for PI/Annexin V binding in CD8^+^ T cells co-incubated for 6 h with total exosomes, MTEX or non-MTEX isolated from plasma of one melanoma patient. Control cells were co-incubated with PBS. In (**b**), % apoptosis of CD8^+^ T cells after co-incubation with exosomes from HDs plasma or total exosomes, MTEX or non-MTEX from melanoma patients’ plasma. In (**c**), % apoptosis of CD8^+^ T cell after co-incubation with total exosomes, MTEX or non-MTEX from melanoma patients in the presence of anti-Fas mAb. Isotype control Ab was used in place of anti-Fas Ab (see SFig. 9b), and all data are normalized to no exosome (PBS) control. Horizontal bars indicate median values. NS: no significant difference. Wilcoxon signed-rank tests were used to evaluate differences between paired samples; Wilcoxon-Mann-Whitney tests were used to evaluate differences between patients and healthy donors.

Exosomes in all three fractions induced significant levels of apoptosis in recipient CD8^+^ T cells (Fig. 5). Apoptosis was exosome dose-dependent (SFig. 9a). MTEX induced apoptosis in 83% of T cells (44% ANXV^+^ and 39% PI + ANXV^+^) in a representative experiment shown in Fig. 5a. Non-MTEX and HDs exosomes induced only low levels of apoptosis (~20% or less) in CD8^+^ T cells. MTEX and total exosomes were strongly apoptotic compared to non-MTEX (*P* = 0.0008) or HDs exosomes (*P* = 0.0009) (Fig. 5b). Apoptosis was partially blocked when neutralizing anti-Fas (ZB4) mAbs were added to co-cultures prior to exosomes (Fig. 5c), and isotype control Abs did not inhibit apoptosis (see SFig. 9b). Exosomes co-incubated with 30 µg Proteinase K and 2.5 mM CaCl_2_ for 1 h had significantly reduced apoptotic activity, and incubation of exosomes at 60 °C for 1 h, eliminated their ability to mediate apoptosis of CD8^+^ T cells (SFig. 9c). Surprisingly, MTEX- induced apoptosis did not significantly correlate with the levels of any single immunoregulatory protein carried by MTEX (data not shown). In contrast, apoptosis mediated by non-MTEX negatively correlated with the levels of CD80 and OX40 expression on these exosomes (respectively, *r* = −0.66, *P* = 0.02, and *r* = −0.63, *P* = 0.03), suggesting that co-stimulatory signals on non-MTEX might ameliorate apoptosis.

#### MTEX-induced NKG2D down-regulation on NK cells

**Figure 6:**
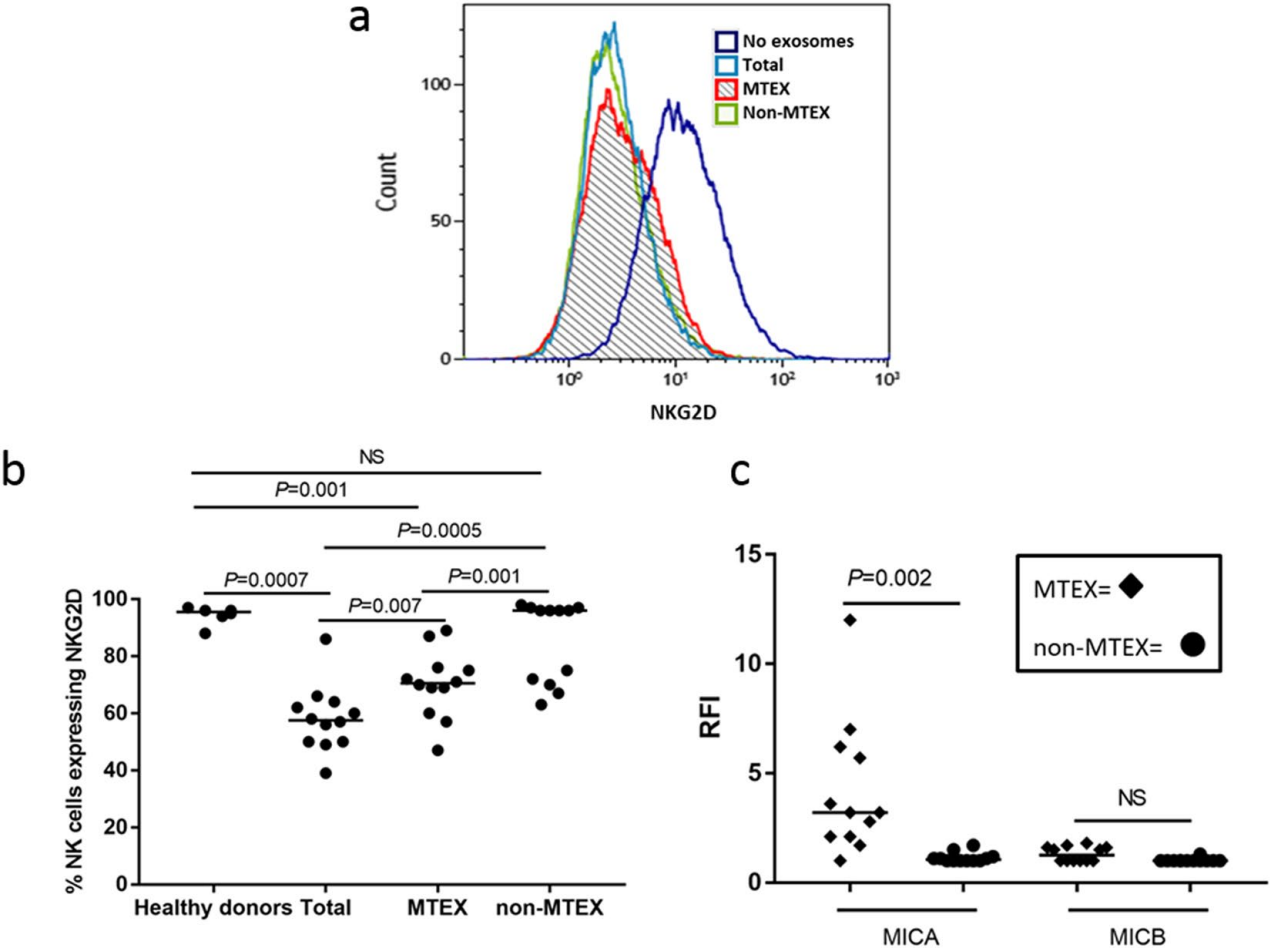
Exosome-mediated effects on NKG2D receptor expression levels on human primary NK cells. In (**a**), total exosomes, MTEX or non-MTEX were co-incubated with human activated NK cells for 24 h. Representative data of exosome-mediated suppression of NKG2D levels by exosomes from one melanoma patient are shown. In (**b**), % of NK cells expressing NKG2D after co-incubation with exosomes of HDs or total exosomes, MTEX or non-MTEX from melanoma patients’ plasma. In (**c**), the presence of MICA or MICB (determined as RFI) on MTEX and non-MTEX. All data were normalized to no exosome (PBS) controls. Blocking of MTEX-mediated downregulation of NKG2D on NK cells by neutralizing anti-TGF-β Ab is shown in SFig. 8. Horizontal bars indicate median values. NS: no significant difference. Wilcoxon signed-rank tests were used to evaluate differences between paired samples; Wilcoxon-Mann-Whitney tests were used to evaluate differences between patients and healthy donors.

Effects of the total exosome, MTEX, non-MTEX and HDs exosomes on primary human NK cells were also investigated. As expected based on our previous work^26,27^, MTEX down-regulated expression levels of NKG2D on NK cells (Fig. 6a,b). Interestingly, total exosomes induced greater NKG2D downregulation than MTEX (*P* = 0.007), perhaps because some non-MTEX also downregulated NKG2D expression levels (Fig. 6b). MTEX were positive for MICA, a ligand for NKG2D, although not for MICB (Fig. 6c) and non-MTEX did not carry MICA or MICB. Thus, NKG2D downregulation by non-MTEX was likely mediated by a mechanism unrelated to MICA or MICB. We showed that blocking with anti-TGF-β mAb restored NKG2D expression on NK cells (SFig. 8b). NKG2B downregulation by non-MTEX correlated with PD1, CD73, and OX40 expression levels (*r* = 0.62, *P* = 0.03; *r* = −0.70, *P* = 0.01; and *r* = 0.70, *P* = 0.01, respectively). However, MTEX-mediated downregulation of NKG2D did not significantly correlate with the expression level of any single immunoregulatory protein carried by MTEX, except for CD80 (*r* = −0.66, *P* = 0.02) (data not shown). Again, the data suggest that NKG2D downregulation may be the result of several different signals simultaneously delivered by MTEX to recipient NK cells.

### Correlations between exosome profiles and exosome functions

Correlations of MTEX and non-MTEX protein profiles with functional changes induced in immune recipient cells by these vesicles were also examined (SFig. 10). Total exosome protein levels in plasma correlated significantly with the MTEX immunosuppressive score (*r* = 0.79, *P* = 0.002), suggesting that higher exosome protein levels in patients’ plasma reflect the enrichment in suppressive MTEX. In fact, apoptosis correlated with the MTEX/total exosome protein ratio (r = 068; *P* = 0.01). The RFI scores for FasL and TRAIL in MTEX were significantly elevated compared with non-MTEX (Fig. 2), accounting for high MTEX-mediated apoptosis. The stim/supp ratio correlated positively with the immunostimulatory score (r = 0.74; P = 0.006).

As expected, non-MTEX-mediated apoptosis was inversely correlated with their stimulatory/suppressive ratio (r = −0.75; *P* = 0.007) and their immunostimulatory score (r = −0.72; *P* = 0.009) (SFig. 8b). Non-MTEX-mediated proliferation correlated positively with their immunostimulatory score (r = 0.59; *P* = 0.04), and inversely with total exosome protein levels (r = −0.59; *P* = 0.046). The non-MTEX stim/supp ratio was inversely correlated with their immunosuppressive score (*r* = −0.76, *P* = 0.004). Non-MTEX-mediated NKG2D downregulation was inversely correlated with their immunosuppressive score (*r* = −0.64, *P* = 0.03) but positively with the MTEX/total exosome protein ratio and the MTEX stim/supp ratio (r = 0.61; *P* = 0.04 and r = 0.81; *P* = 0.002, respectively). In aggregate, these results linking immune activities of MTEX or non-MTEX with their phenotypic characteristics emphasize: (a) superior immune suppressive activity of MTEX over non-MTEX; (b) dependence of exosome-mediated suppression/stimulation of immune cells on the profile of immunoregulatory proteins carried by these exosomes; and (c) the ability of both MTEX and non-MTEX to alter functions of immune cells depending on the stim/supp protein ratios they carry.

### Correlations of exosome profiles with patients’ clinicopathological data

We explored associations between exosome molecular profiles and the available clinicopathologic variables (SFig. 10) to determine whether the observed MTEX and non-MTEX characteristics could be used as potential markers of disease status or activity. Although not powered for a formal correlative assessment, the study provided several potentially instructive insights. For example, the finding that MTEX-mediated apoptosis of CD8+ T cells did not correlate with disease status or stage, while non-MTEX ability to induce apoptosis associated with disease stage (r = 0.61, *P* = 0.04) reveals an unexpected potential of non-MTEX to serve as a correlate of melanoma progression. A significant inverse correlation of the stim/supp ratio with disease stage (r = −0.83, *P* = 0.0007) (SFig. 5b) emphasizes the importance of using this ratio rather than individual components of the exosome cargo in future correlative studies. Although disease status at blood draw was inversely correlated with the expression level of PDL-1 on MTEX (*r* = −0.62, *P* = 0.03), it did not correlate with any of the other proteins evaluated in MTEX or non-MTEX (data not shown). Age at blood draw and age at diagnosis both correlated with the non-MTEX PD1 level (at diagnosis: *r* = 0.59, *P* = 0.045; at blood draw: *r* = 0.61, *P* = 0.04), but not with any of the other individual protein levels (data not shown). Tentatively, this correlation links age with PD1-mediated suppression in melanoma.

## Discussion

Early evidence suggested that EVs isolated from supernatants of murine or human melanoma cell lines were decorated by death ligands such as FasL, TRAIL or biologically-active TGF-β1, and directly or indirectly suppressed functions of various immune cell subsets^8,9,28–32^. Recent studies confirmed that exosomes obtained from melanoma patients’ plasma contained immunosuppressive proteins, including PD-L1^4,33^. However, the cellular source of immunosuppressive exosomes in patients’ plasma has remained unknown. In this communication, we demonstrate for the first time that MTEX isolated from melanoma patients’ plasma are largely responsible for immune suppression previously observed with plasma-derived exosomes. Immunoaffinity-based separation of exosome subsets using mAbs specific for the CSPG4 epitopes into MTEX and non-MTEX allowed for examination of the cargos as well as functions of MTEX and unequivocally demonstrated MTEX capacity to mediate immunosuppression.

To examine the immunoregulatory “exosome profile” we selected a handful of proteins that are known to play a key role in immunoregulation^34^ and are present in MTEX^11,35^. Although MTEX were enriched in inhibitory proteins and mediated the bulk of suppressive activity, non-MTEX were not entirely devoid of suppressor functions. HDs exosomes also mediated low levels of immune suppression. These findings are not unexpected given the similarity of molecular cargo that all exosomes carry. Differences in the cargo components between MTEX and non-MTEX were *quantitative* and were highly significant. The mean stim/supp ratio was 0.6 for MTEX versus 1.4 for non-MTEX and 2.2 for HDs exosomes. Thus, it was the disparity in MTEX/total exosomes ratios or stim/supp ratios, and not expression levels of individual stimulatory or inhibitory proteins, that discriminated between MTEX and non-MTEX. The paucity in MTEX of co-stimulatory ligands, especially CD40L and OX40L (both members of the TNF superfamily of proteins critical for interactions with recipient immune cells^36,37^) and the enrichment in levels of inhibitory ligands contribute to significantly greater MTEX-mediated immunosuppression. The enrichment of stimulatory proteins in non-MTEX counterbalances the effects of inhibitory ligands that non-MTEX also co-express and favors lymphocyte stimulation. This suggests that the sum of inhibitory vs stimulatory proteins on the exosome surface determines the distinct functional potentials of MTEX and non-MTEX. It is of interest to note that the content of immunoregulatory proteins in MTEX versus non-MTEX is reminiscent of that in tumor cells, which are highly enriched in immunoinhibitory factors compared to normal cells^38^.

The mechanistic aspects of MTEX interactions with recipient immune cells were also addressed by our studies. We previously showed that primary T cells activated via the T-cell receptor only minimally internalize PKH26-labeled exosomes even after prolonged (72 h) co-incubation^25^. In contrast, labeled TEX were detected in the cytoplasm of NK cells after 6 h co-incubation^39^. Further, we and others have reported that TEX-induced immunosuppression involves signaling of FasL^+^ exosomes via CD95 (Fas) on activated CD8^+^ T cells^13,28,30^. In this study, MTEX carrying FasL induced apoptosis of >50% of activated T cells within 6 h of co-incubation, and anti-Fas Abs significantly, albeit not completely, inhibited T-cell apoptosis (Fig. 5; SFig. 9b). These data indicate the inhibitory signals delivered by MTEX to the cognate receptors on the recipient cell surface are sufficient for eliciting changes in the phenotypic or functional profiles of recipient T cells without vesicle internalization. Co-incubation of MTEX with activated T cells for 6 h also reduced CD69 expression on the T-cell surface. Simultaneous downregulation of CD69 proteins on T cells and changes of the mRNA transcript for CD69 in these cells (SFig. 6a) suggest that the MTEX signal delivered to the cell surface leads to transcriptional activation. Furthermore, MTEX induced the NF-κB pathway activation in T cells after 30 min of co-incubation (SFig. 6b). The recovery of immune cell functions in the blocking experiments performed with MAbs or pharmacological inhibitors of PD-L1, TGF-β, FasL, and NF-κB activation indicates that these proteins participate in MTEX-induced suppression of T cell or NK cell functions (SFig. 8) as also reported for TEX produced by tumor cell lines^22,27,28,39,40^.

MTEX are present in excess in the tumor microenvironment (TME). MTEX surface is decorated with multiple inhibitory proteins (Fig. 2c), MTEX circulate freely in all body fluids and can deliver multiple inhibitory signals to T cells, ***simultaneously*** activating various inhibitory molecular pathways and resulting in T-cell dysfunction or death. The stim/supp protein ratio in MTEX and the MTEX/total exosome ratio in plasma are characteristic features of each melanoma patient and determine MTEX immunoinhibitory functions^41,42^. Our data support the concept of MTEX carrying an excess of suppressive ligands as a component of the powerful tumor-driven system of immune suppression in melanoma. We have previously suggested that this model of exosomes simultaneously delivering multiple suppressive signals to immune cells in cancer represents an underestimated barrier to successful cancer immunotherapy^39^.

In principle, the distinct phenotypic and functional characteristics of MTEX and non-MTEX could be linked to disease activity and progression. A recent report showed that melanoma exosomes carrying PD-L1 suppress functions of CD8^+^ T cells and facilitate tumor growth^4^. Increases in PD-L1^+^ exosomes during early stages of anti-PD-1 therapy discriminated clinical responders from non-responders, suggesting that circulating exosomes isolated from plasma based on PD-L1 expression predicted responses to anti-PD-1 therapy in melanoma^4^. PD-L1 expression levels on MTEX or non-MTEX in our study were variable and did not correlate with their ability to reduce CD69 expression on T cells. However, anti-PD1 mAb significantly blocked CD8^+^ T cell proliferation inhibition by MTEX, emphasizing the significance of the PD-1/PD-L1 pathway in exosome-mediated immune suppression. We have also identified an association between PD-L1 levels on MTEX and non-MTEX with NKG2D downregulation in NK cells. Our previous reports attributed TEX-mediated downregulation of the NKG2D receptor on human NK cells to the TGF-β-mediated suppression^27,39^. Here, we showed that although MTEX carry MICA, an inhibitory ligand for NKG2D^43^, the TGF-β pathway emerges as another inhibitory mechanism of NKG2D downregulation of NK cells. Our findings support the view of MTEX as carriers of multiple biologically-active inhibitory signals with the potential to *in tandem* activate several inhibitory molecular pathways in recipient immune cells.

This study was not designed to assess correlations of experimental data with clinical endpoints. Nevertheless, Spearman’s correlations identified a few significant associations of the data with disease status or stage. Confirming the relevance of apoptosis to the exosome cargo^44^, the non-MTEX stim/supp ratio was negatively associated with MTEX-mediated apoptosis. Interestingly, a significant positive correlation between T-cell apoptosis in non-MTEX and disease stage was identified. While the biological or clinical usefulness of this association remains unclear, it might be taken as a sign that in high-stage disease, non-MTEX become reprogrammed by the tumor, acquiring the ability to mediate apoptosis. In this cohort of patients of with advanced metastatic disease, such reprogramming of normal cells could be expected, as suggested by our previous studies of T cell-derived CD3^+^ exosomes in patients with cancer^45^.

This study is the first to show that MTEX, a subset of exosomes derived from melanoma cells and abundantly present in plasma of melanoma patients, are largely responsible for immune suppression in melanoma that potentially promotes tumor immune escape and tumor progression. To further define the role of MTEX in melanoma progression and confirm their clinical significance, future studies with much larger patient cohorts, including patients with primary and metastatic disease, will be necessary and are in progress in our laboratory.

## Supplementary information

Supplementary Information 

## Data Availability

Additional data are summarized and are included as supplementary materials available at the *Scientific Report*’s website.
