# Supplementary material for: Melanoma cell-derived exosomes in plasma of melanoma patients suppress functions of immune effector cells
Source: Sci Rep. 2020 Jan 9;10:92. doi: 10.1038/s41598-019-56542-4 (PMC6952363; doi:10.1038/s41598-019-56542-4)
Supplement: Supplementary file 1 — Supplementary Information [file 41598_2019_56542_MOESM1_ESM.pdf]

Supplementary data

Melanoma cell-derived exosomes in plasma of melanoma patients suppress functions of immune effector cells

Priyanka Sharma[1], Brenda Diergaarde[2], Soldano Ferrone[3], John M. Kirkwood[4], Theresa L. Whiteside[1,5 *]

[1]Department of Pathology, University of Pittsburgh School of Medicine and UPMC Hillman Cancer Center, Pittsburgh, PA, 15213, USA

[2]Department of Human Genetics, Graduate School of Public Health, University of Pittsburgh and UPMC Hillman Cancer Center, Pittsburgh, PA 15213, USA

[3]Department of Surgery, Massachusetts General Hospital and Harvard Medical School, Boston, MA 02114

[4]Department of Medicine, University of Pittsburgh School of Medicine and UPMC Hillman Cancer, Pittsburgh, PA, 15213, USA

[5]Departments of Immunology and Otolaryngology, University of Pittsburgh School of Medicine, Pittsburgh, PA, 15213, USA

Running title: MTEX and non-MTEX in melanoma patients' plasma

*** Corresponding author:** Theresa L. Whiteside, PhD
University of Pittsburgh School of Medicine
UPMC Hillman Cancer Center
5117 Centre Avenue, Suite 1.27
Pittsburgh, PA 15213
Phone: (412) 624-0096
FAX: (412) 624-0264
email: whitesidetl@upmc.edu

Supplementary Methods

Antibodies used

The following Abs were purchased from commercial vendors: anti-CD63 (353004, clone H5C6), anti-gp100 (312204, clone HI10a) and anti-VLA4 (304304, clone 9F10), anti-CD39 (328206, clone A1), anti-CD73 (344004, clone AD2), anti-Fas-L (306407, clone NOK-1), anti-TRAIL (308210, clone RIK-2) and anti-CTLA-4 (349908, clone L3D10) Abs were from Biolegend (San Diego, CA, USA). Anti-Melan A (sc-20032, clone A103) Ab was from Santa Cruz Biotechnology (Dallas, TX, USA). Anti-Fas (555673, clone DX2) and anti-HLA-DR (555811, clone G46-6) Abs were from BD Pharmingen (San Jose, CA, USA). Anti-PD-1 (1202799-42, clone eBioJ105) and anti-PDL-1 (48-5983-42, clone MIH1) Abs were from eBioscience (San Diego, CA, USA). Anti-LAP-TGFβ1 (FAB2463, clone 27232), anti-TYRP2 (370504, clone GM26E7) and anti-MICB (clone #236511) Abs were from R&D Systems (Minneapolis, MN, USA). Anti-CD40 (clone#5C3 RUO) and anti-CD80 (clone #L307.4) Abs were from BD Pharmingen (San Jose, CA, USA). Anti-CD40L (clone#24-31) and anti-OX-40L (clone #11C3.1) Abs were from Biolegend (San Diego, CA, USA). Anti-OX40 (clone #ACT35) Ab was from eBioscience (San Diego, CA, USA). Antibody biotinylation kits were purchased from Novus Biologicals (Littleton, CO, USA), The ExoCap™ Streptavidin Kit was from MBL International Corporation (Woburn, MA, USA), and the Annexin V-FITC Apoptosis detection kit and CFSE Fluorescent Cell Labeling kit were from Abcam.

Exosome protein detection

Titration experiments were performed individually for every Ab to determine optimal concentrations for detection of the relevant antigens. Reproducibility of the flow cytometry-based detection assay was validated using exosomes isolated from plasma of 3 different melanoma patients, each split into 3 aliquots each tested in 3 parallel detection assays. Minimal intra-individual variability was 7% and inter-individual differences were > 93%.

Functional Studies with Exosomes

All co-cultures were set up with $CD8^+$ T cells isolated from normal human peripheral blood using AutoMACs Pro Separator as described [25]. All media used for co-cultures were supplemented with 10% FBS (v/v) depleted of bovine exosomes by centrifugation at 100 000 x g overnight.

Functional Assays

(a) CD69 downregulation on T cells by exosomes

T cells were activated for 6h at 37ºC with CD3/CD28 T-cell activator (25µl/ml, Stemcell, Vancouver, BC, Canada) and IL-2 (150IU/mL, PeproTech, Rocky Hill, CT, USA) in freshly-prepared RPMI 1640 medium. Expression of CD69 on the T-cell surface was evaluated by flow cytometry before and after co-incubation of activated $CD8^+$ T cells ($2x10^5$ /well) with exosomes at the concentration of 10µg protein for 16h at 37ºC in a CO_2 incubator. Co-cultures containing PBS only served as controls.

Changes in mRNA transcripts for CD69 in $CD8^+$ T cells co-incubated with exosomes were also measured. T cells were first activated with anti-CD3/anti-CD28 beads as described above for 6 h at 37ºC. T cell aliquots ($2x10^5$) were co-incubated with MTEX, non-MTEX or total exosomes (10µg protein each) for 6h at 37ºC. PBS served as "no exosomes" control. After co-incubation, cells were lysed in 350µL RNA lysis buffer (EXIQON kit). Complementary DNA (cDNA) was synthesized from 100ng of total RNA using a Qiagen polymerase chain reaction kit. Briefly, template DNA, primer solutions, dNTP mix, 5X Qiagen One-step RT-PCR buffer, and RNase-free water were thawed and placed on ice. Template RNA was added to the reaction mixture in the individual PCR tubes. cDNA was amplified using 36 cycles in a thermal cycler (Col Parmer, IL, USA) with primers for CD69 and β-actin as follows: reverse transcription 30min at 50ºC; initial PCR activation 15min at 95ºC; denaturing 1min at 94ºC; annealing 1 min at 65ºC; extension 1 min at 72ºC. The final extension lasted 10 min at 72ºC. The amplified sequences were resolved on a 2% agarose gel electrophoresis and visualized using 0.1% ethidium bromide under UV light.

Specific primers used:

CD69 5`-CATAGCTCTCATTGCCTTATCAGT-3` (forward primer)

5`-CCTCTCTACCTGCGTATCGTTT-3` (reverse primer)

β-actin 5` GTGGGGCGCCCCAGGCACCA-3` (forward primer)

5` CTCCTTAATGTCACGCACGATTTC-3` (reverse primer)

(b) NFkB activation in $CD8^+$ T cells co-incubated with exosomes

CD8$^+$ T cells were co-incubated with total plasma exosomes, MTEX, non-MTEX or PBS for 30min. T cells were washed with PBS and fixed using 4% (w/v) paraformaldehyde in PBS for 20min and then permeabilized with 0.1% Triton X for 10min. After incubation with anti-NFkB p65 Abs (Abcam;1:500) overnight at 4°C, cells were washed 2x and incubated with the Alexa Fluor 488-labeled secondary Abs (Abcam:1:500) for 1h at RT. Cells were also stained with Red Fluorescent Phalloidin F-actin Abs (Abcam) for 30min in the dark. Cells were then incubated with the Hoechst dye (1:1,000) for 5min and mounted for microscopy using Prolong Gold solution (Thermo Fisher Scientific Invitrogen, # P10144). Imaging was performed using the Carl Zeiss LSM 880 confocal microscope at fixed settings across treatments.

(c) Apoptosis of CD8$^+$ T cells co-incubated with exosomes

CD8$^+$ T cells were plated in wells of a 96-well plate at the concentration of $2x10^5$ cells/well and activated as described above for 12-24 h at 37ºC. Exosomes (10µg protein/50uL PBS) were added to cells and co-incubated for 6h at 37ºC. Co-cultures containing no exosomes served as controls. Apoptosis was measured using AnnexinV-FITC Apoptosis Detection kit (Abcam, MA, USA) by flow cytometry. Anti-Fas neutralizing antibody (10µg/mL) or IgG1 isotype control (Millipore Sigma) was added to activated CD8$^+$ T cells and incubated for 1h before co-incubation with exosomes to block T-cell apoptosis.

(d) CFSE-based proliferation assays

$CD8^+$ T cells were labeled with 1.5μM CFSE (Abcam) in 0.1% BSA in PBS (w/v) for 10min at 37°C. Staining was quenched with an equal volume of exosome-depleted FBS [25]. CFSE-labeled $CD8^+$ T cells ($1x10^5$cells/well) were activated with CD3/28 beads (T-Cell Activation Kit, Miltenyi) at the cell to bead ratio of 1:1 for 24h at 37^0C. T cells were co-incubated with exosomes (10μg protein/50μL PBS) for 3d at 37°C in a CO_2 incubator. Proliferation of T cells was measured on day 4 by flow cytometry, and the data were analyzed by Kaluza and Modfit software (Verity Software House). Percentage suppression of proliferation in co-cultures with exosomes was calculated as previously described by us [25] and compared to control T cells incubated without exosomes.

(d) NKG2D down-regulation on NK cells

NK cells were isolated from PBMCs obtained from healthy donors using AutoMACS and an NK cell isolation kit (Miltenyi). NK cells were placed in RPMI1640 medium containing IL-2 (150 IU/mL and IL-15 (20ng/mL) and were co-incubated with or without exosomes for 24h as previously described [22]. Percentages of NK cells with downregulated NKG2D expression levels (MFI) were measured by flow cytometry with gating on $CD3^-CD56^+$ NK cells. Activated NK cells incubated without exosomes served as controls.

Blocking experiments

Human $CD8^+$ T cells were isolated and activated with anti-CD2/anti-CD3/anti-CD28 beads (130-091-441, Miltenyi, San Diego, CA, USA) at the 1:2 beads to cell ratio in the presence of IL-2 (150 U/mL, Peprotech) overnight (10^5 cells/well of a 96-well plate) in RPMI 1640 10%. After $CD8^+$ T cells were exposed to ILL16 (200 nmol, 2539, Tocris),

APCP (100 µg, AF1086 lot #ICA02, R&D Sytems), anti-Fas Ab (10µg, 05-338 lot #2896737 cloneZB4, Millipore), neutralizing TGF-β Ab (IDII, 10 nM) and appropriate isotype controls were added 1 h before the addition of MTEX. Next freshly isolated MTEX (5 µg) were added to the triplicate wells and co-incubated with T cells for 72 h. Cultures without MTEX (PBS only) served as controls. Cell numbers were measured with an Accuri flow cytometer (BD Biosciences, San Jose, CA USA).

Supplementary Figures

SFigure 1a-f: Characteristics of total exosomes isolated by mini-SEC from plasma of melanoma patients or healthy donors. In **(a)**, transmission electron microscopy of total exosomes in the miniSEC fractions #4 in a patient with melanoma (**left**) or a HD (**right**). In **(b)**, representative qNano profiles of exosomes from a melanoma patient and a healthy donor to illustrate their respective sizes and numbers of nanoparticles per mL of plasma. In (**c**), Western blots of total exosomes (10ug exosome protein/lane) from plasma of melanoma patients or healthy donors to show that all carry TSG101, suggesting an origin from the endocytic compartment of parent cells. Note that patients' exosomes are enriched in FasL which is consistent with their ability to mediate apoptosis of activated human T cells. Exosomes from plasma of healthy donors carry little or no FasL. In **(d)** comparisons of Western blots and on-bead flow cytometry results for several immunosuppressive proteins in MTEX and non-MTEX isolated from plasma of two melanoma patients. For Western blots, each lane was loaded with 10ug exosome protein. The flow cytometry data are shown as relative fluorescence intensity (RFI) values. See Methods for details. In (**e**) transmission of electron microscopy of **non-MTEX** separated from exosomes in fraction #4 by immunocapture and removal of MTEX. In **(f)** on-bead flow cytometry of MTEX which are CSPG4+ but CD3neg.

Sfigure 1a

Exosomes from melanoma patient's plasma

Exosomes from healthy donor's plasma

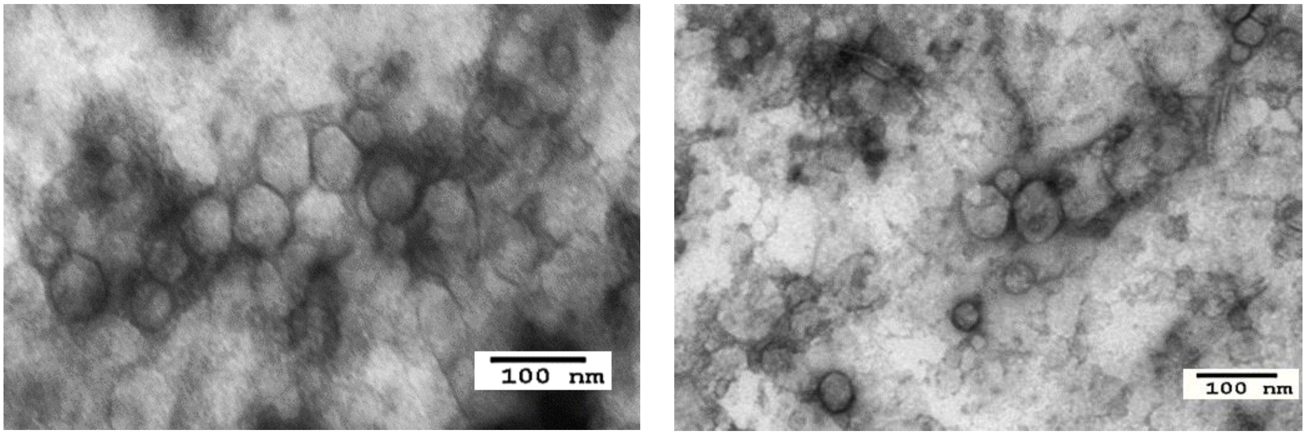

Sfigure 1b

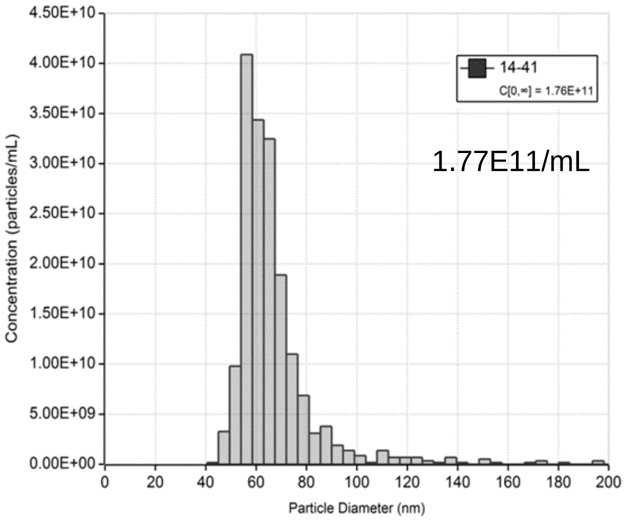

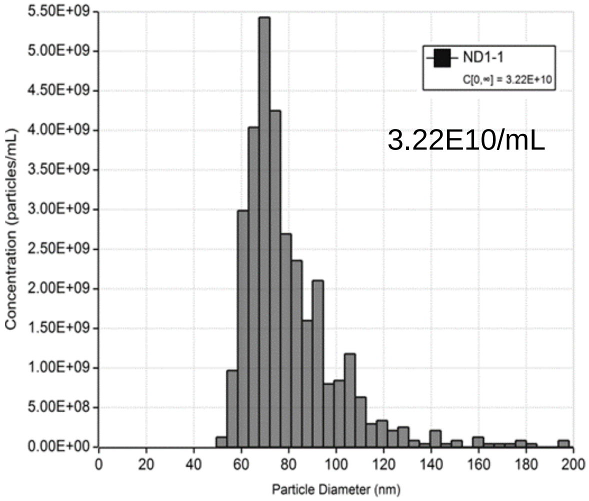

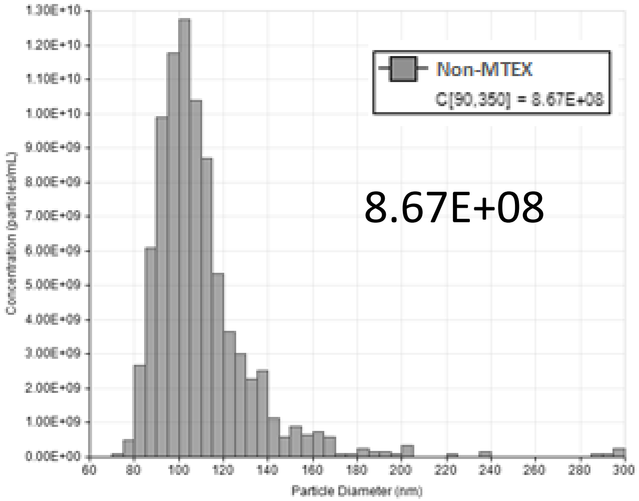

Sfigure 1c

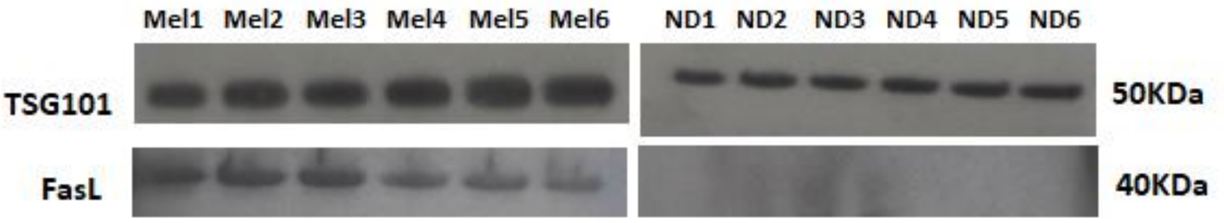

Sfigure 1d

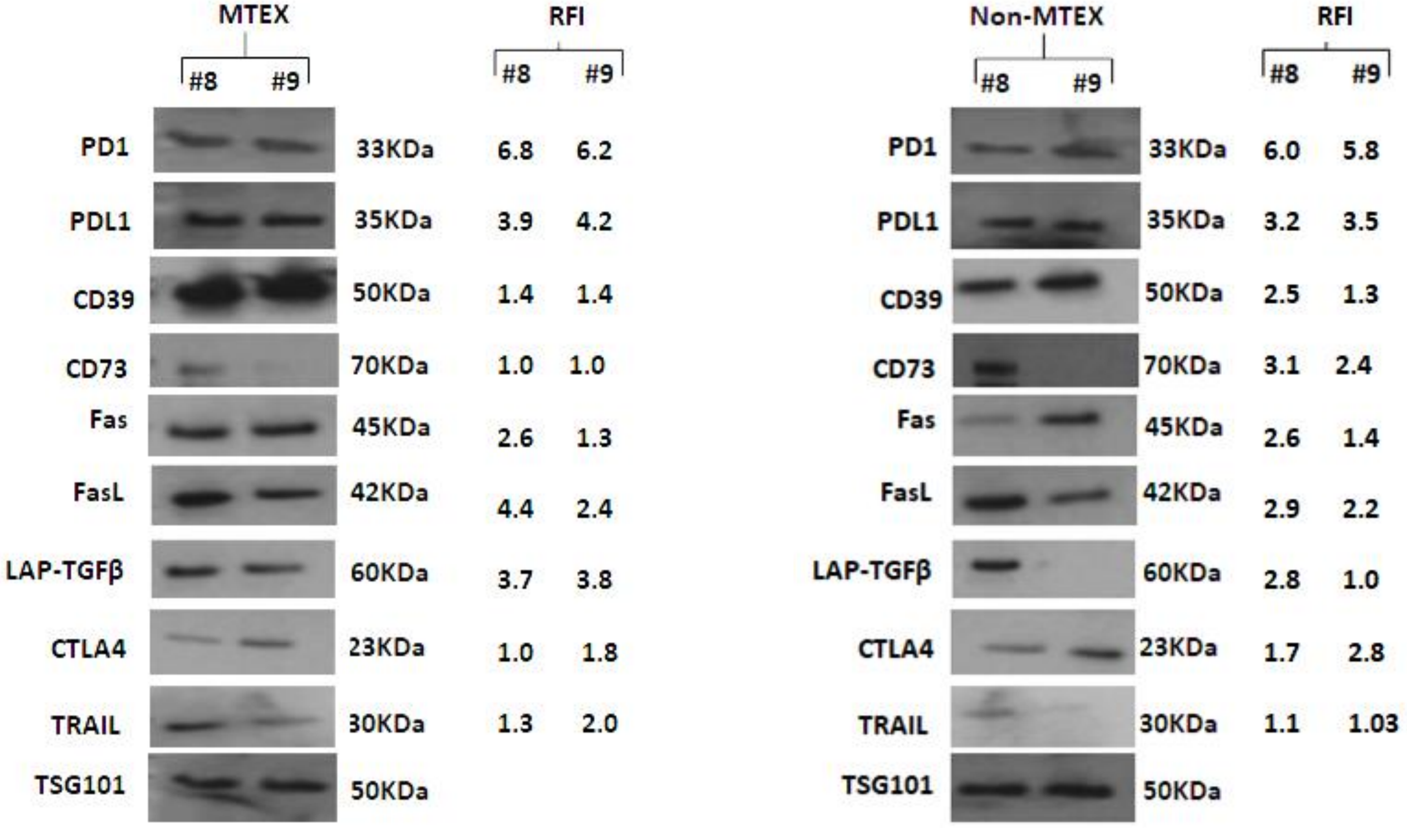

Figure 1e

TEM of non-MTEX isolated from a melanoma patient plasma

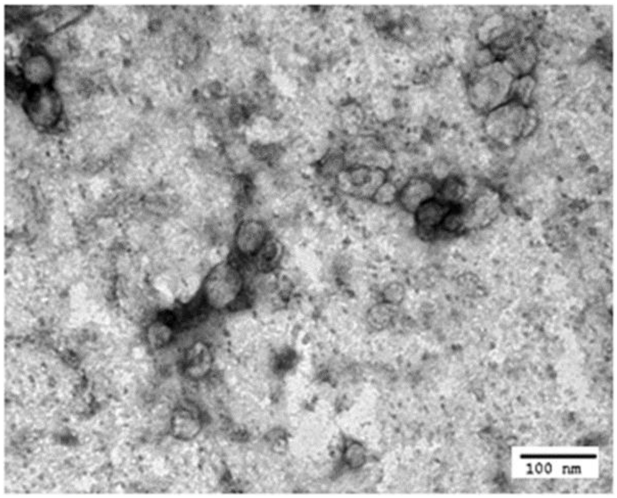

Figure 1f CD3 expression on MTEX captured with anti-CSPG4

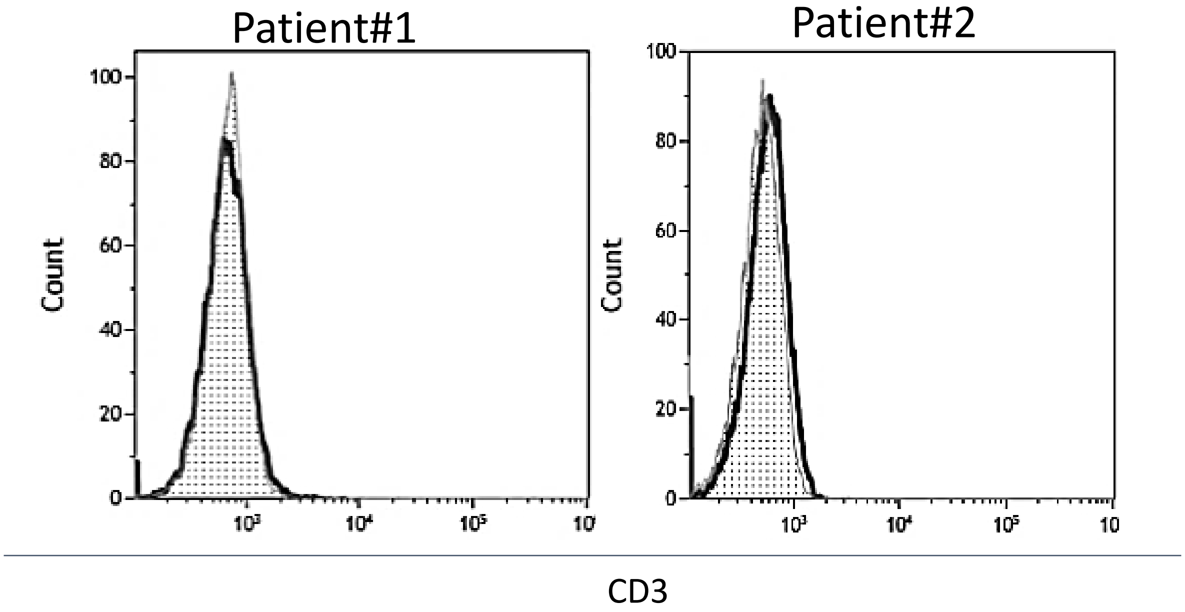

Sfigure 2a: In **(a)**, CSPG4 expression on MTEX captured with anti-CSPG4 mAb (clone 763.74) and detected with a fluorochrome-labeled anti-CSPG4 mAb (clone 225.28). **Nearly all MTEX are CSPG4+.**

(a)

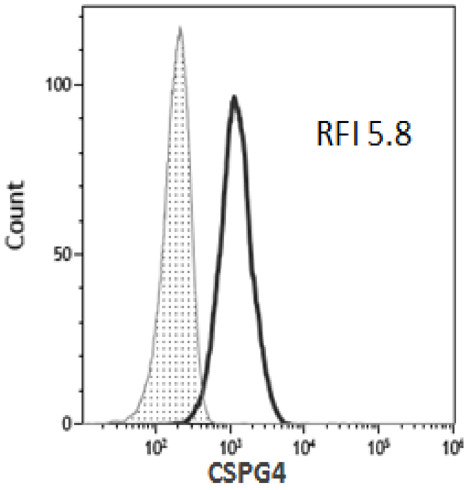

Sfigure 2b: On-bead flow cytometry results for expression of CSPG4 on MTEX and non-MTEX. Exosomes were captured with beads coated with biotinylated anti-CSPG4 mAbs (left) or biotinylated anti-CD63 mAb (right). **No CSPG4 was detected on non-MTEX**. For detection of CSPG4 on the exosome surface, a different clone of fluorescently labeled antiCSPG4 mAb was used. Shaded peaks = isotype control Abs. RFI=MFI of detection mAb/MFI of isotype control mAb.

(b)

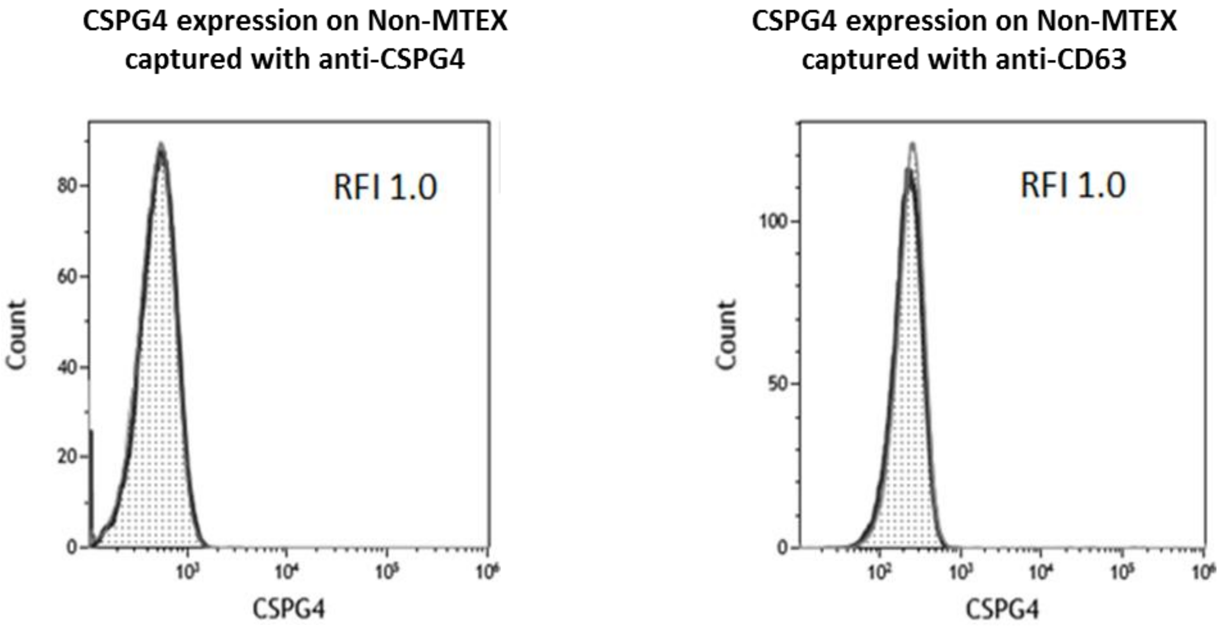

Sfigure 2c: CSPG4 was not detected on exosomes isolated from plasma of healthy donors. Flow cytometry was performed as described in the legend to SFigure 2a

CSPG4 expression on exosomes from healthy donor

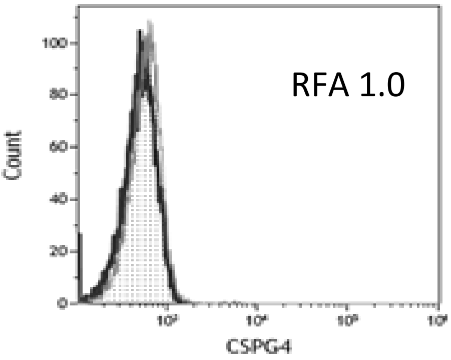

SFigure 2d: CD3 is expressed only on the surface of non-MTEX indicating that most non-MTEX are derived from T cells. Non-MTEX were negative for platelet antigens (e.g. CD61 or IIb/3a)

(d)

Patient #1 (Non-MTEX)

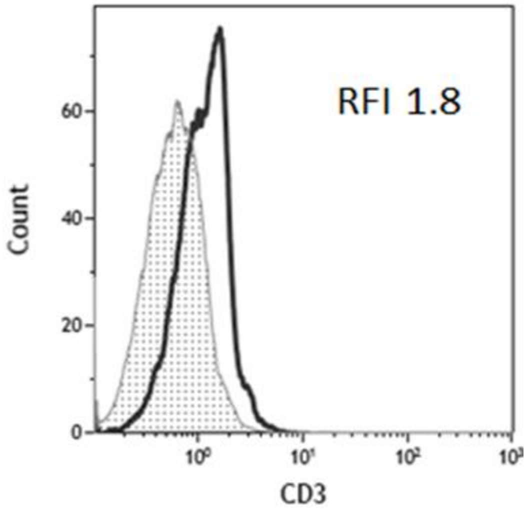

Patient #2 (non-MTEX

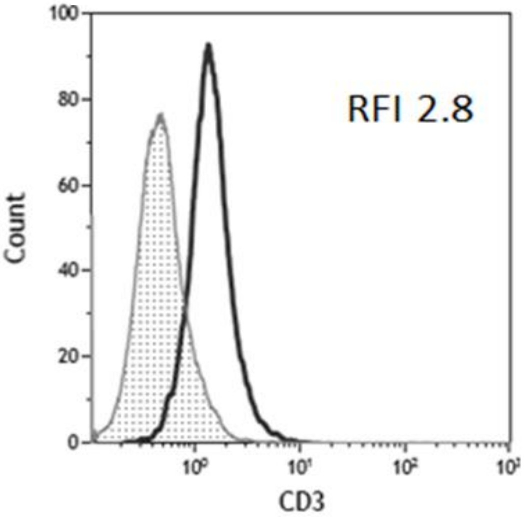

SFigure 3

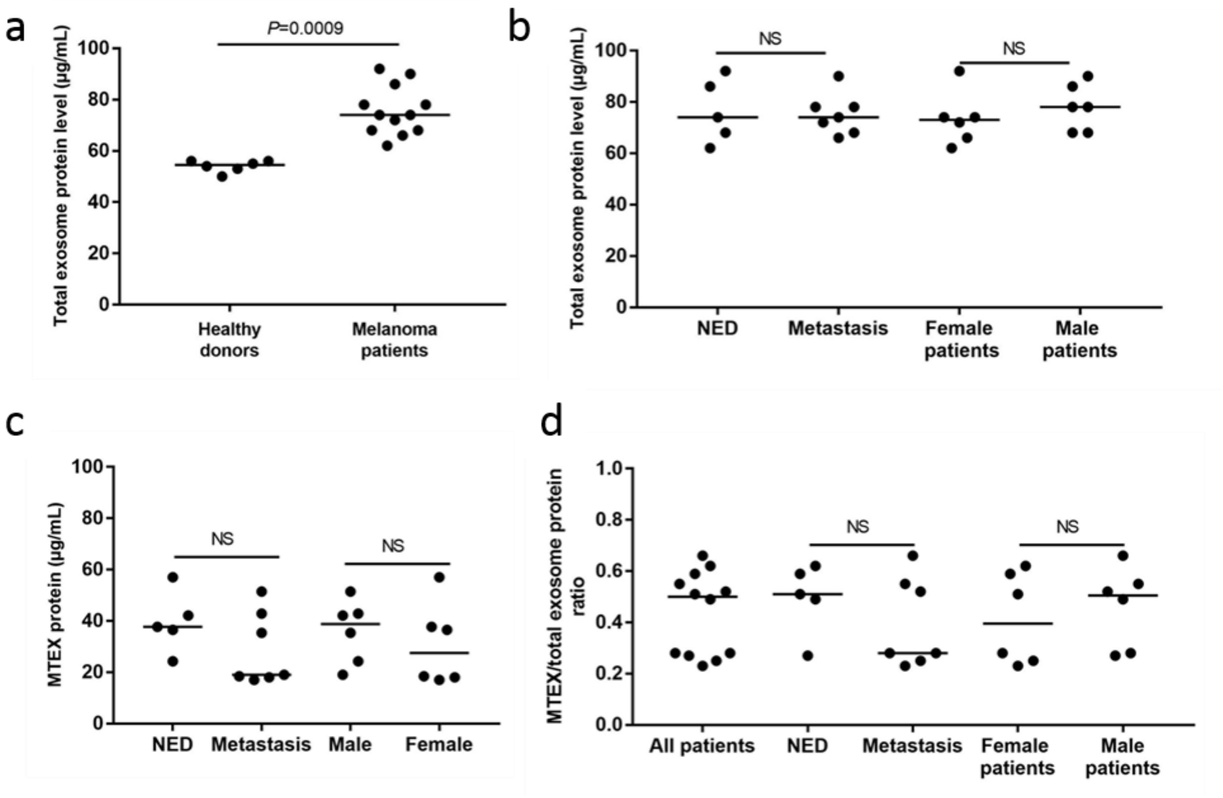

SFigure 3: Exosome protein levels. In **(a)**, total exosome protein levels in plasma of healthy donors and melanoma patients. In **(b)**, total exosome protein levels by disease status and sex. In **(c)**, MTEX protein levels by disease status and sex. In **(d)**, the MTEX/total exosome protein ratio, for all patients, and by disease status and sex. Wilcoxon-Mann-Whitney tests were used to evaluate differences between the different groups. Horizontal bars indicate median values. NS: no significant difference.

SFigure 4 a-c: Levels of MAAs and immunoregulatory proteins on MTEX and non-MTEX determined by on-bead flow cytometry of captured and non-captured exosomes. Representative results of quantitative flow cytometry for exosomes are shown (data for patient #8 are shown). In **(a)**, the data for MAAs; in **(b)** for immunosuppressive proteins; and in **(c)** for immunostimulatory proteins.

SFigure 4a

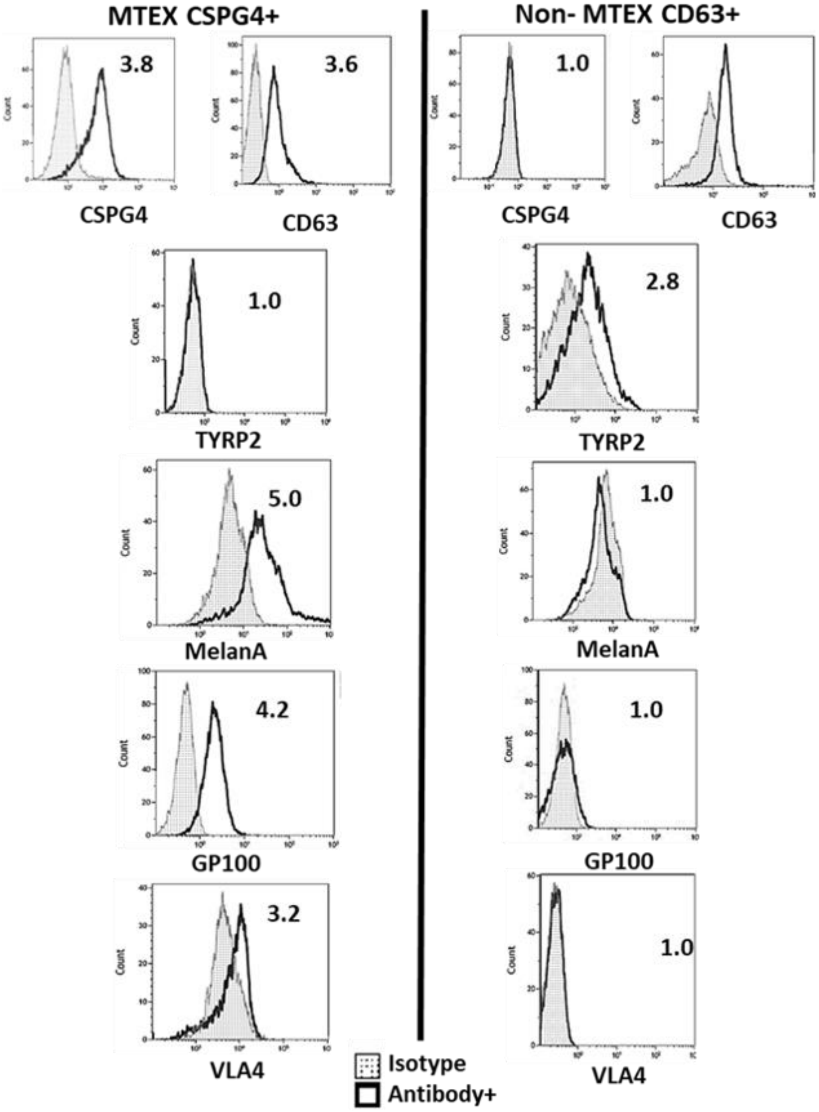

SFigure 4b

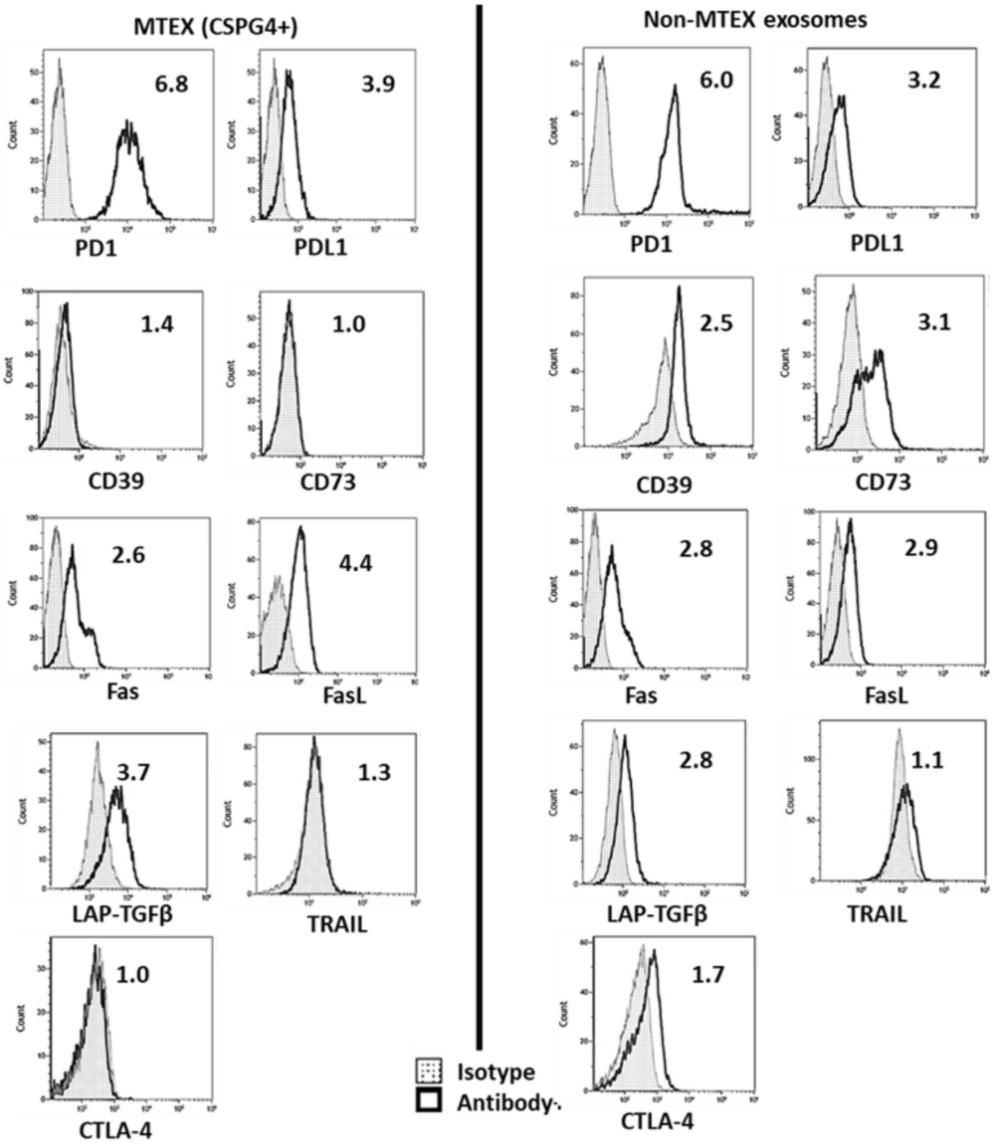

MTEX (CSPG4+)
Non-MTEX exosomes
6.8
PD1
3.9
PDL1
6.0
PD1
3.2
PDL1
1.4
CD39
1.0
CD73
2.5
CD39
3.1
CD73
2.6
Fas
4.4
FasL
2.8
Fas
2.9
FasL
3.7
LAP-TGFβ
1.3
TRAIL
2.8
LAP-TGFβ
1.1
TRAIL
1.0
CTLA-4
1.7
CTLA-4
Isotype
Antibody
Count

SFigure 4c

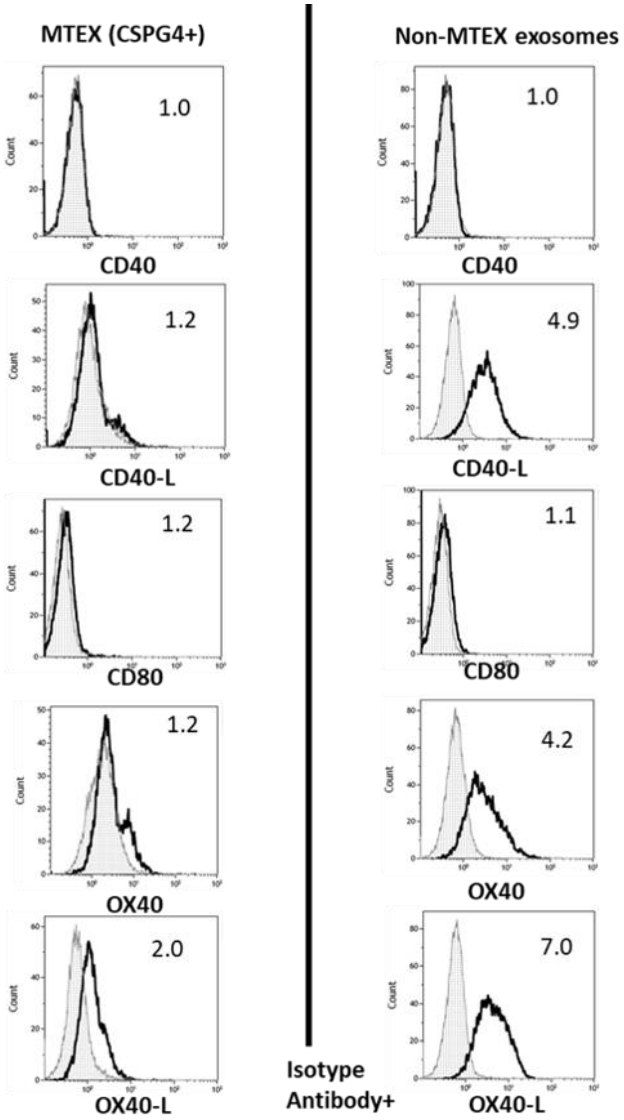
MTEX (CSPG4+)
Non-MTEX exosomes
1.0
CD40
1.0
CD40
1.2
CD40-L
4.9
CD40-L
1.2
CD80
1.1
CD80
1.2
OX40
4.2
OX40
2.0
OX40-L
7.0
OX40-L
Isotype
Antibody+
Count

SFigure 5: Correlations between individual proteins, for MTEX and non-MTEX.

(a) MTEX

	PD1	PDL-1	CD39	CD73	Fas	FasL	LAP-TGFβ	TRAIL	CTLA-4	CD40	CD40L	CD80	OX40	OX40L
**PD1**	1													
**PDL-1**		1		-0.59 *P* =0.04								0.63 *P* =0.03		
**CD39**			1											
**CD73**		-0.59 *P* =0.04		1										
**Fas**					1								0.67 *P* =0.02	0.68 *P* =0.01
**FasL**						1								
**LAP-TGFβ**							1							
**TRAIL**								1						
**CTLA-4**									1					
**CD40**										1				
**CD40L**											1			
**CD80**		0.63 *P* =0.03										1		
**OX40**					0.67 *P* =0.02								1	0.60 *P* =0.04
**OX40L**					0.68 *P* =0.01								0.60 *P* =0.04	1

b) Non-MTEX

	PD1	PDL-1	CD39	CD73	Fas	FasL	LAP-TGFβ	TRAIL	CTLA-4	CD40	CD40L	CD80	OX40	OX40L
**PD1**	1											0.61 *P* =0.04		
**PDL-1**		1							0.74 *P* =0.006					
**CD39**			1	0.72 *P* =0.008			0.66 *P* =0.02				0.65 *P* =0.02			
**CD73**			0.72 *P* =0.008	1							0.79 *P* =0.002			
**Fas**					1									
**FasL**						1						-0.59 *P* =0.04		
**LAP-TGFβ**			0.66 *P* =0.02				1		-0.66 *P* =0.02					
**TRAIL**								1						
**CTLA-4**		0.74 *P* =0.006					-0.66 *P* =0.02		1					
**CD40**										1				
**CD40L**			0.65 *P* =0.02	0.79 *P* =0.002							1			
**CD80**	0.61 *P* =0.04					-0.59 *P* =0.04						1	0.69 *P* =0.01	
**OX40**												0.69 *P* =0.01	1	
**OX40L**														1

Spearman's correlation coefficients were calculated. Only significant correlations (P <0.05) are shown; blue=positive and orange=negative correlation

SFigure 6a, b

SFigure 6a

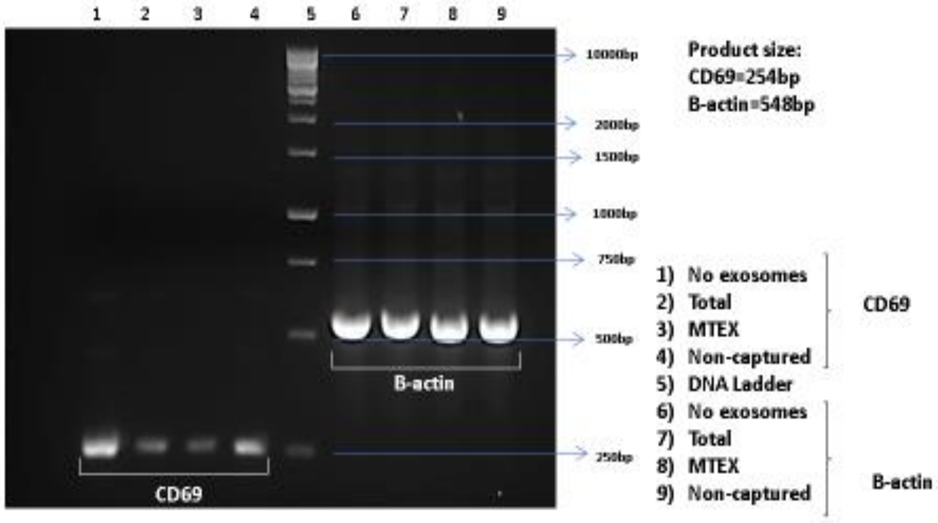

Sfigure 6a: RT-PCR of activated human primary CD8⁺ T cells co-incubated with MTEX, non-MTEX or total exosomes obtained from plasma of a melanoma patient. Cells were either left untreated (PBS instead of exosomes) or co-incubated with total exosomes, captured exosomes (MTEX) or non-captured exosomes (non-MTEX) from plasma of a melanoma patient for 6h. Note a decrease in CD69 transcript in T cells co-incubated with MTEX or total melanoma exosomes but not with non-MTEX. cDNA was synthesized from 100ng of total RNA using a polymerase chain reaction kit (Qiagen) as described in Methods.

SFigure 6b

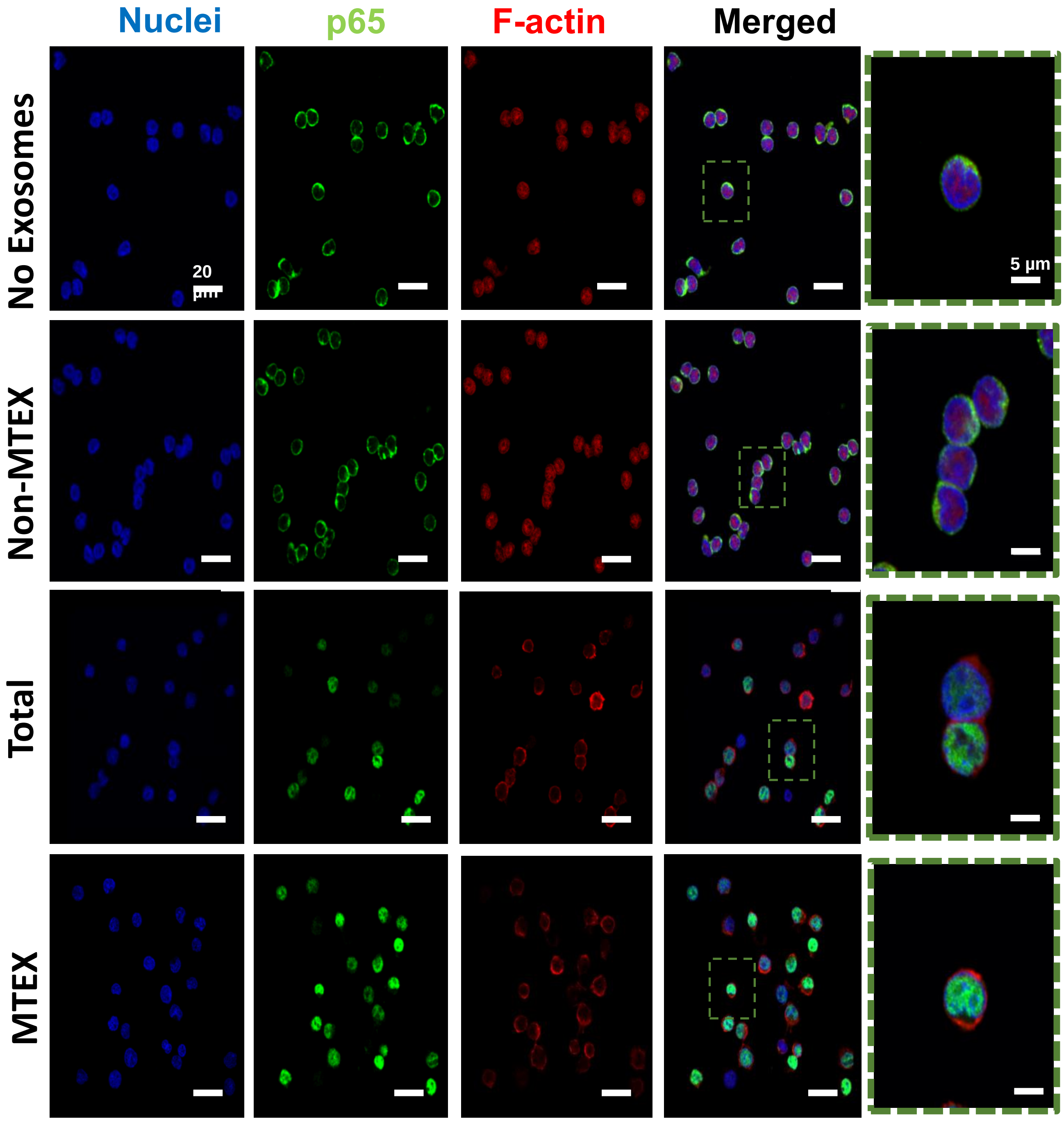

Sfigure 6b: Confocal microscopy for NFkB activation following coincubation of human promary CD8^{+} T cells with exosomes for 30 min. Nuclear translocation of p65 to the nucleus in total melanoma exosomes and in MTEX ndicates activation of NFkB. No p65 translocation is seen after co-incubation of T cells with non-MTEX. Courtesy of Saigopalakrishna Yerneni.

SFigure 7 Blocking of MTEX-induced CD69 downregulation with anti-PD1 mAB. CD69 expression after block with anti-PD1 blocking antibody. $CD8^+$ cells were isolated from blood or healthy donor and activated as described in Methods. The cells were co-incubated with Total exosomes, MTEX, non-MTEX or no exosomes in the presence of anti-PD1 mAb or go to IgG isotype control Ab for 16h.

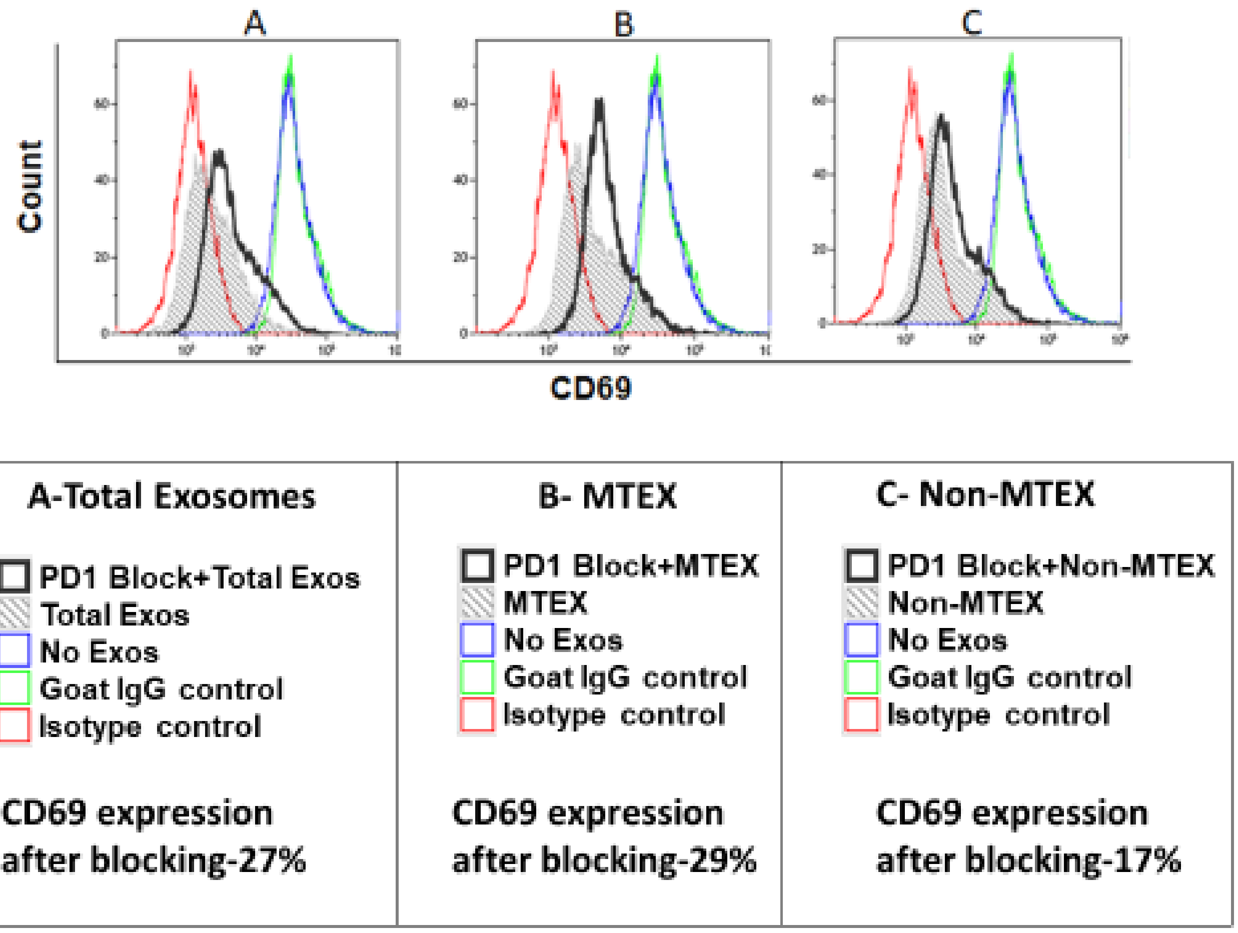

SFigure 8a.

Blocking of MTEX-induced suppression of CD8⁺ T-cell proliferation

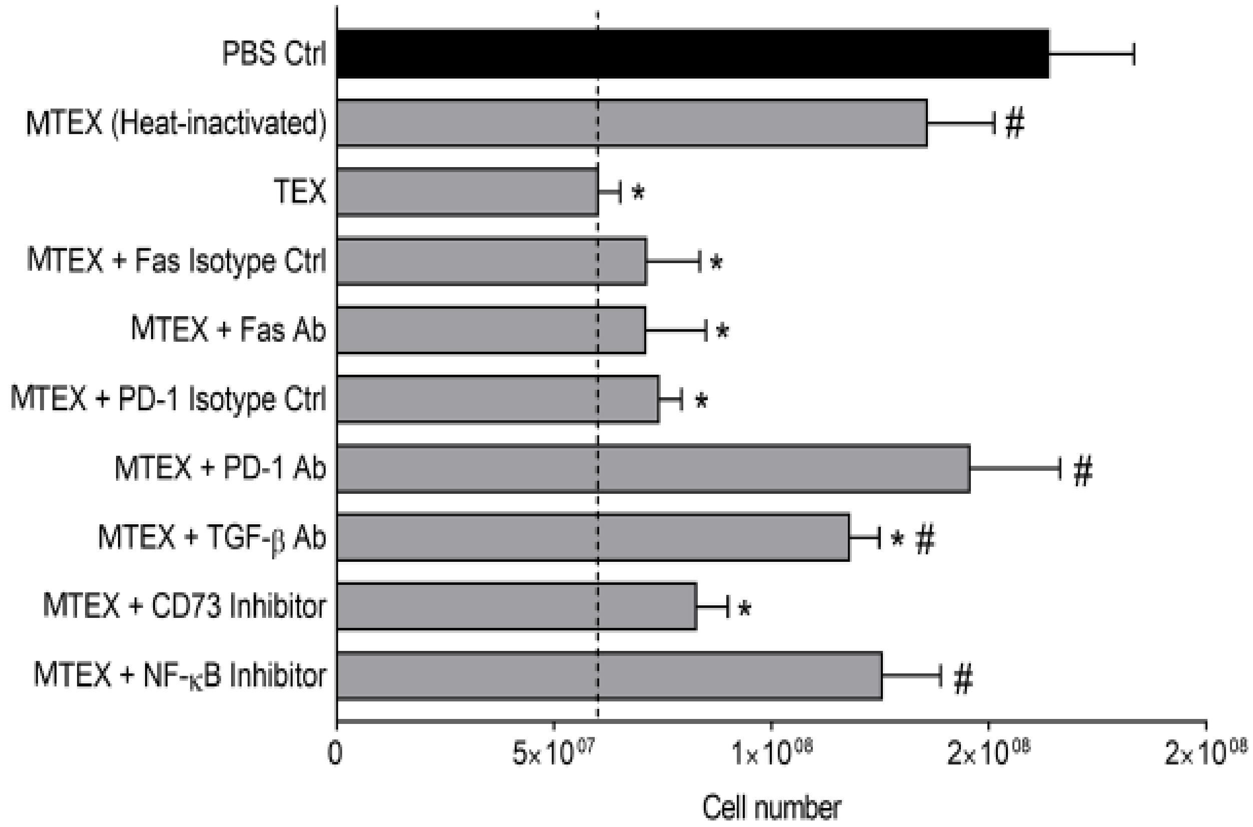

Human primary CD8⁺ T cells were isolated, activated with anti-CD3/CD28 beads in the presence of IL-2 for 18h and then incubated with blocking agents or isotype controls for 1h followed by 72h co-incubation with MTEX . Cell numbers were counted using a flow cytometer. The dotted line indicates the level of MTEX-induced suppression of T-cell proliferation. Data are means ± SD of triplicate measures. *$p<0.05$ vs PBS control(no MTEX) and #$p<0.05$ vs MTEX alone.

SFigure 8b.

Reversal of MTEX-induced suppression of NKG2D expression level on human NK cells by neutralizing anti-TGF-β antibody

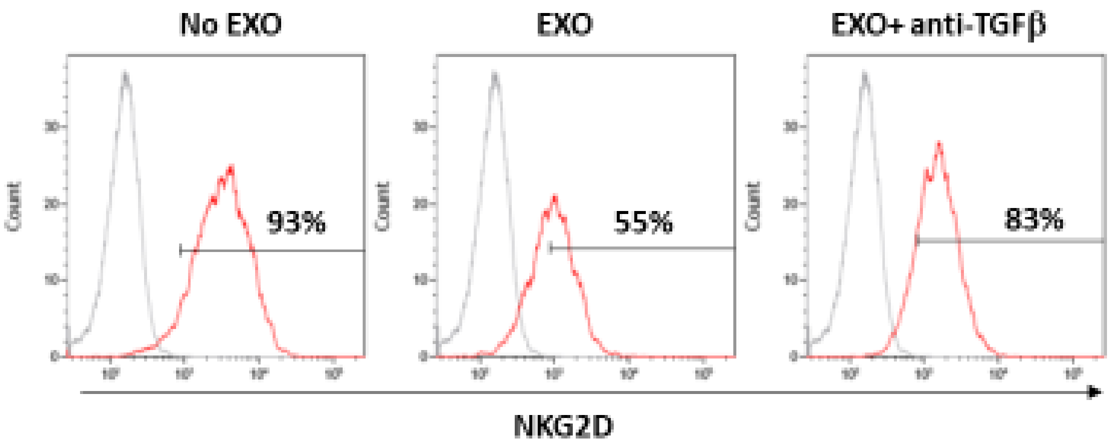

NK92 cells (100,000 cells) were incubated for 48hrs (no exosomes), with Mel526 exosomes (10ug) or with Mel526 exosomes (10ug) plus anti-TGFb antibody (clone 1D11: 10nM), then stained with PE conjugated anti-NKG2D antibody. Isotype-PE (gray)

SFigure 9 a-c

SFigure 9a

Apoptosis assay with human primary CD8$^+$ T cells: Dose response for exosomes obtained from a melanoma patient's plasma

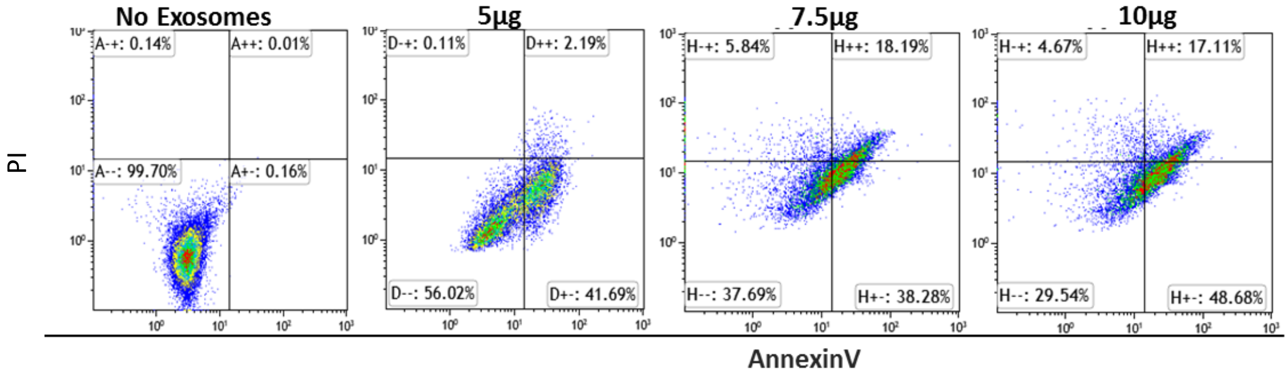

Data were acquired after 6h co-incubation as described in Methods.

SFigure 9b

Inhibition of MTEX induced apoptosis in the presence of anti-Fas Abs (data shown in Figure 5c) or isotype Abs control for this experiment.

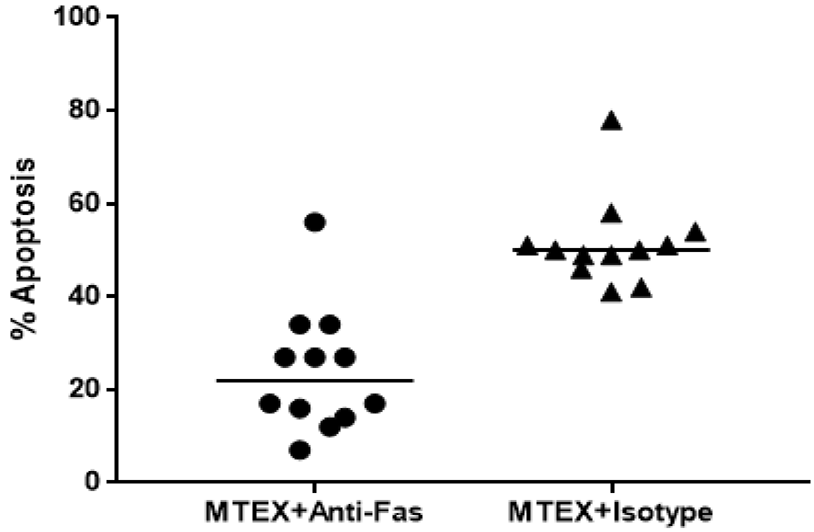

MTEX of the one outlier patient induced very high apoptosis (80%) in T cells and anti-Fas Abs at the concentration used only partially lowered the apoptosis level.

SFigure 9c

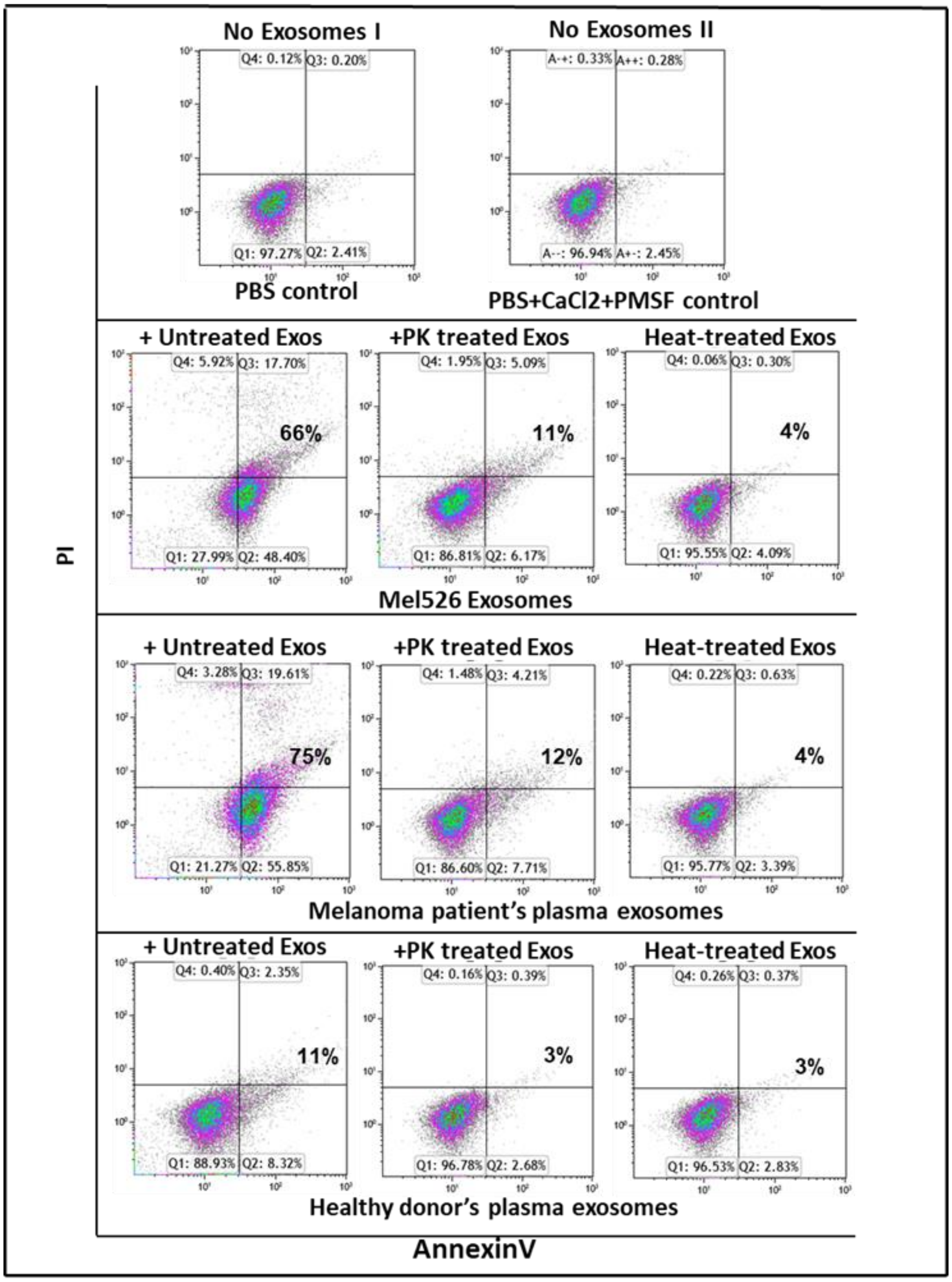

SFigure 9c: Effects of proteinase K (PK) or heat (60°C) on exosome-mediated apoptosis of $CD8^+$ T cells. Column 1 shows apoptosis mediated by untreated exosomes; column 2 shows apoptosis mediated by PK-treated exosomes; column 3 show apoptosis mediated by heat-treated exosomes. T cells (2×10^5) were coincubated with exosomes (10µg protein) for 6h. The percentages of T-cell apoptosis for each co-incubation experiment are indicated.

SFigure 10: Correlations between exosome profiles or functions and clinical variables.

(a) MTEX

	Total protein	MTEX/Total protein ratio	Supp score	Stim score	Stim/supp ratio	CD8+ prolif	CD69	NKG2D	% Apop	Age at dx	Age at draw	Stage	Sex	Disease status
Total protein	1		0.79<br>*P* =0.002											
MTEX/Total protein ratio		1							0.68<br>*P* =0.01					
Supp score	0.79<br>*P* =0.002		1											
Stim score				1	0.74<br>*P* =0.006									
Stim/supp ratio				0.74<br>*P* =0.006	1									
CD8+ prolif						1								
CD69							1							
NKG2D								1						
% Apop		0.68<br>*P* =0.01							1					
Age at dx										1	0.84<br>*P* =0.0006		0.63<br>*P* =0.03	
Age at draw										0.84<br>*P* =0.0006	1			
Stage												1		
Sex										0.63<br>*P* =0.03			1	
Disease status														1

(b) Non-MTEX

	Total protein	MTEX/Total protein ratio	Supp score	Stim score	Stim/supp ratio	CD8+ prolif	CD69	NKG2D	% Apop	Age at dx	Age at draw	Stage	Sex	Disease status
Total protein	1					-0.59<br>*P* =0.046								
MTEX/Total protein ratio		1						0.61<br>*P* =0.04						
Supp score			1		-0.76<br>*P* =0.005			-0.64<br>*P* =0.03						
Stim score				1		0.59<br>*P* =0.04			-0.72<br>*P* =0.009					
Stim/supp ratio			-0.76<br>*P* =0.005		1			0.81<br>*P* =0.002	-0.73<br>*P* =0.007			-0.83<br>*P* =0.0007		
CD8+ prolif	-0.59<br>*P* =0.046			0.59<br>*P* =0.04		1								
CD69							1							
NKG2D		0.61<br>*P* =0.04	-0.64<br>*P* =0.03		0.81<br>*P* =0.002			1						
% Apop				-0.72<br>*P* =0.009	-0.73<br>*P* =0.007				1			0.61<br>*P* =0.04		
Age at dx										1	0.84<br>*P* =0.0006		0.63<br>*P* =0.03	
Age at draw										0.84<br>*P* =0.0006	1			
Stage					-0.83<br>*P* =0.0007				0.61<br>*P* =0.04			1		
Sex										0.63<br>*P* =0.03			1	
Disease status														1

Spearman's correlation coefficients were calculated. Only significant correlations (P <0.05) are shown; blue=positive and orange=negative correlation

SFigure 11

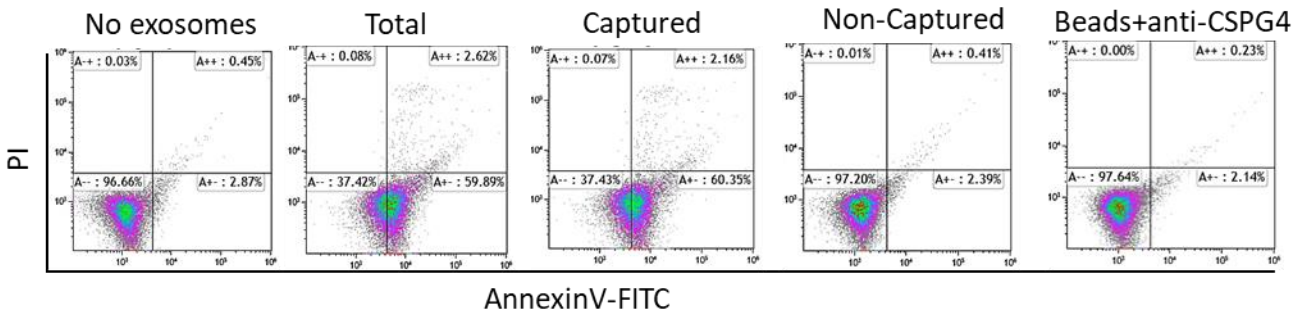

SFigure 11: Evidence that the use of MTEX captured on beads does not interfere with measurements of T cell functions. Shown is apoptosis of primary activated $CD8^+$ T cells co-incubated with **(a)** no exosomes (PBS); **(b)** total plasma exosomes (no beads); **(c)** MTEX (captured on beads); **(d)** non-MTEX (no beads); **(e)** beads coated with anti-CSPG4 Ab used for MTEX capture (no exosomes). Exosomes were added to activated T cells at the concentration of 10μg protein; 6h co-incubation was followed by flow cytometry. Apoptosis was measured using Annexin V-FITC Apoptosis Detection kit (Abcam).

SFigure 12: Whole Western Blot Scan (a-d): Additional supplementary whole blot scans for SFigure 1d

(a)

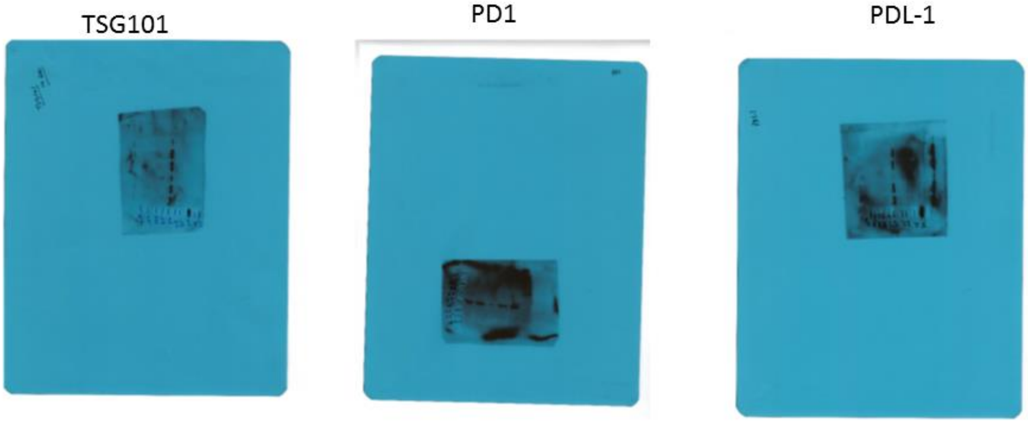

(b)

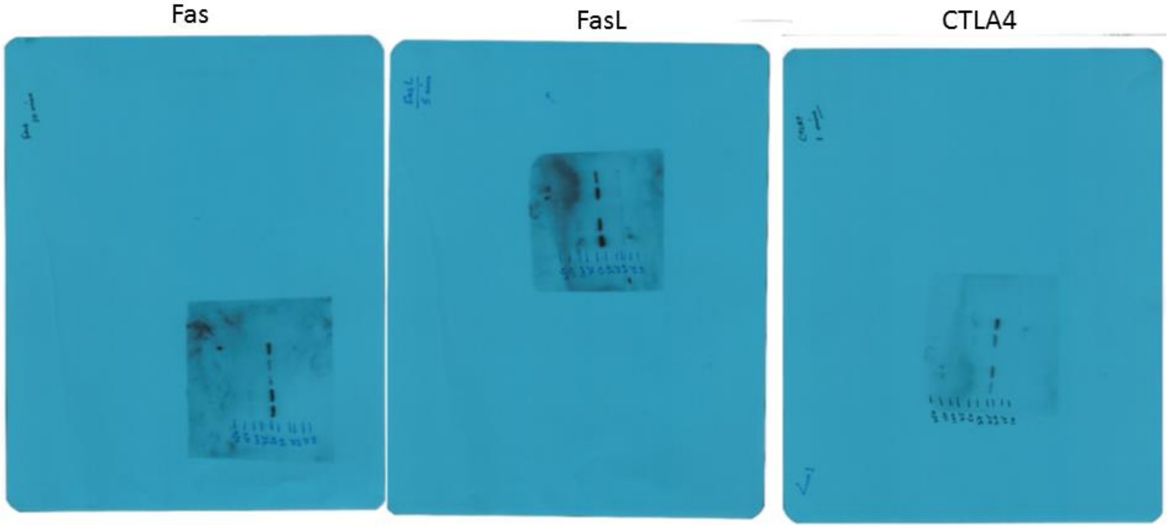

(c)

CD39

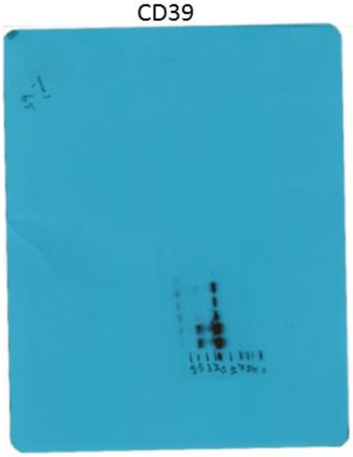

CD73

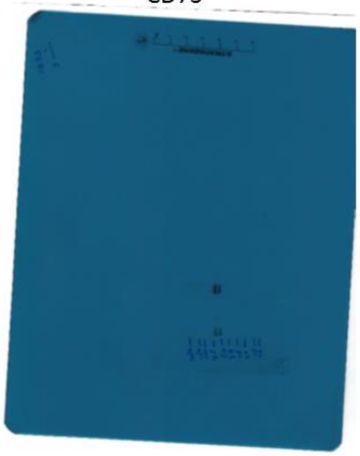

(d)

LAP-TGFβ

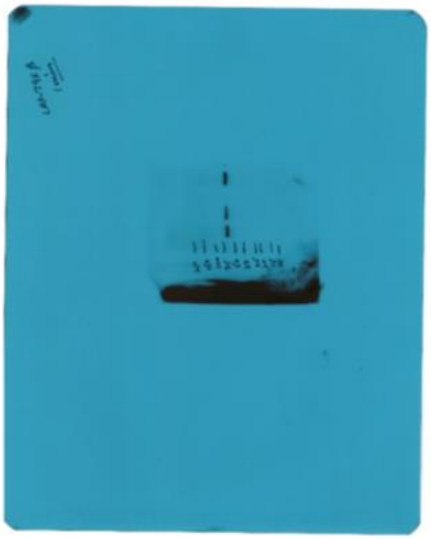

TSG101

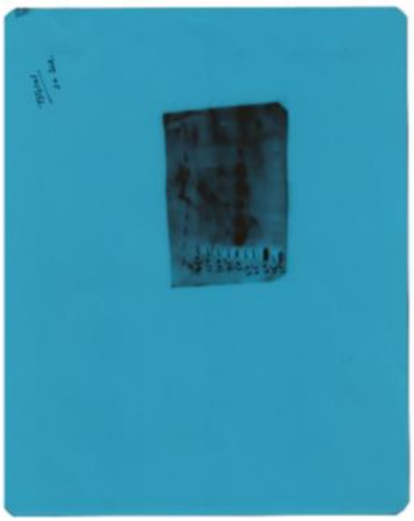

STable 1. Selected characteristics of the study population

Subject #	Status	Gender	Age at Diagnosis	Age at blood draw	Stage at diagnosis	Disease status at blood draw	Total exosomes protein levels (µg/mL)
1	case	Male	69	70	1A	NED[b]	86
2	case	Female	38	41	1A	metastasis	66
3	case	Female	39	39	1B	NED	62
4	case	Female	32	32	1B	NED	92
5	case	Female	55	72	1B	NED	74
6	case	Male	80	86	2A	metastasis	68
7	case	Male	78	82	2B	metastasis	78
8	case	Male	69	69	2B	NED	90
9	case	Female	66	66	3B	metastasis	74
10	case	Male	53	53	3C	metastasis	68
11	case	Female	69	69	IV	metastasis	72
12	case	Male	67	71	Ocular melanoma	metastasis	78
13	control	Male	na[a]	72	na	na	56
14	control	Female	na	72	na	na	50
15	control	Female	na	68	na	na	53
16	control	Male	na	66	na	na	54
17	control	Female	na	69	na	na	56
18	control	Female	na	68	na	na	55

[a]na: not applicable; [b]NED: no evidence of disease

MTEX/total exosome protein ratios. Controls: mean age at blood draw in years (± sd): 69.2 (± 2.4), median: 68.5, range: 66-72). Age at blood draw did not differ between cases and controls (*P*=0.78).

STable 2a. Results of on-bead flow cytometry analysis of proteins carried by MTEX

Melanoma associated antigens (MAA)[a]

Patient	MAA RFI score[a]	CSPG4	TRYP2	MelanA	Gp100	VLA4
1	13	2.2	2	3.8	1.6	3.4
2	12	4.6	2.5	2.8	1	1.1
3	14.8	5.4	2.6	4.8	1	1
4	14.6	4.8	3.1	3.8	1	1.9
5	19	4.6	5.2	5.4	2	1.8
6	12.7	2.3	2.3	2	3.2	2.9
7	14.3	5.8	1.8	3	2.2	1.5
8	17.2	3.8	1	5	4.2	3.2
9	10.8	4.2	1	1	3.6	1
10	15.3	4.3	3.8	2.5	1.5	3.2
11	7	1.6	1.6	1.4	1.2	1.2
12	12.2	4.1	3.1	2.1	1.4	1.5

[a] MAA RFI score = sum of CSPG4, TRYP2, MelanA, Gp100, and VLA4

Mean RFI Score for MAA: **13.6**

Immunosuppressive proteins

Patient	Supp RFI Score[b]	PD1	PDL-1	CD39	CD73	Fas	FasL	LAP-TGFβ	TRAIL	CTLA-4
1	16.8	3.3	4	1	1.2	4	1.8	4	3.8	1
2	13.4	1.6	1	1.8	2.4	2	2.2	1	3.8	1.2
3	13	1.1	2.5	3	1	1	2	1	1	2.5
4	20.3	8	1.6	1.1	1	4.8	7.2	1.8	6	1.6
5	15.2	2.8	1.8	2	1	2.3	2.8	3	3.6	1
6	10.8	5.9	1	1	2.9	2.3	2.3	1	1	1.6
7	19.9	2	1.2	1.5	1	2.8	4.8	4.8	4	2.6
8	16.7	6.8	3.9	1.4	1	2.6	4.4	3.7	1.3	1
9	17.1	6.2	4.2	1.4	1	1.3	2.4	3.8	2.5	1.8
10	9.8	5.8	1.3	1.3	1	5	2.4	1	1	1.8
11	15.5	6.5	1.4	2.7	2.2	2.5	2.8	1.8	3.6	1
12	15.2	6.1	1	3.4	1.4	2.4	2.3	1.3	3.2	2.6

[b] Supp RFI score: the sum of PDL-1, CD39, CD73, FasL, LAP-TGFβ, TRAIL, and CTLA-4

Mean RFI Score for immunosuppressive proteins: **15.3**

Immunostimulatory proteins

Patient	Stim RFI score[c]	CD40	CD40L	CD80	OX40	OX40L
1	14.4	1	1	3	4	5.4
2	5.3	1	1.1	1	1.1	1.1
3	6.2	1	1.1	1	1.1	2
4	12	1	4.2	1	3.5	2.3
5	6.4	1	1	1	1	2.4
6	6	1	1.1	1	1.4	1.5
7	10.7	1	1	1	3.8	3.9
8	6.6	1	1.2	1.2	1.2	2
9	7.2	1	1	1.2	1.9	2.1
10	14.6	1	2.2	1.2	3	7.2
11	8.9	1	1.1	1	3.7	2.1
12	7.2	1	1	1	1.7	2.5

[c] Stim RFI score: the sum of CD40, CD40L, CD80, OX40, and OX40L

Mean RFI score for immunostimulatory proteins: **8.8**

Mean Stim/Supp ratio (is immunostimulatory RFI score/immunosuppressive RFI score) for MTEX: **0.6**

STable 2b. Results of on-bead flow cytometry analysis of proteins carried by non-MTEX

Melanoma associated antigens (MAA)

Patient	MAA RFI score[a]	CSPG4	TRYP2	MelanA	Gp100	VLA4
1	5	1	1	1	1	1
2	6.3	1	2.3	1	1	1
3	5	1	1	1	1	1
4	5.4	1	1.4	1	1	1
5	5	1	1	1	1	1
6	5.3	1	1	1	1.3	1
7	5	1	1	1	1	1
8	6.8	1	2.8	1	1	1
9	5.4	1	1	1	1.4	1.02
10	5	1	1	1	1	1
11	5.1	1	1.1	1	1	1
12	5.9	1	1.1	1.8	1	1

[a] MAA RFI score: the sum of CSPG4, TRYP2, MelanA, Gp100, and VLA4

Mean RFI Score for MAA: **5.4**

Immunosuppressive proteins

Patient	Supp RFI score[b]	PD1	PDL-1	CD39	CD73	Fas	FasL	LAP-TGFβ	TRAIL	CTLA-4
1	10.1	11	2.5	1.8	1	1.9	1	1	1	1.8
2	8.1	5.8	1.3	1	1	1	1	1.4	1.4	1
3	11.7	10	1.5	2	1	2.8	1.8	2	1.6	1.8
4	8.4	5.2	1.5	1	1	1	1	1	1	1.9
5	11.8	9.8	1.4	1	1	4.2	2.8	1	3.5	1.1
6	14.49	9	1	2.8	3.08	1.1	1	3.53	2.08	1
7	9.2	13	2.2	1	1	1	1	2	1	1
8	17.3	6	3.2	2.5	3.1	2.8	2.9	2.8	1.1	1.7
9	14.23	5.8	3.5	1.3	2.4	1.4	2.2	1	1.03	2.8
10	10.14	5.6	2.9	1	1	4.2	1.06	1	1.08	2.1
11	13.31	6.2	1.3	3.1	2.5	1.8	1.08	2.51	1.42	1.4
12	11.81	6	1.02	1.4	1	3.8	3.2	3.19	1	1

[b] Supp RFI score: the sum of PDL-1, CD39, CD73, FasL, LAP-TGFβ, TRAIL, and CTLA-4

Mean RFI score for immunosuppressive proteins: **11.7**

Immunostimulatory proteins

Patient	Stim RFI score[c]	CD40	CD40L	CD80	OX40	OX40L
1	20.1	1	3.4	2.3	6.6	6.8
2	16.4	1	3.2	1.7	4.7	5.8
3	22.1	1	2.8	1.8	7	9.5
4	11.4	1	1.6	1	3.8	4
5	16.9	1	1.8	1	3.1	10
6	18.9	1	3.8	2.1	4.5	7.5
7	16.6	1	2	1.8	7.2	4.6
8	18.2	1	4.9	1.1	4.2	7
9	15.9	1	4.5	1.1	2	7.3
10	12.5	1.4	3.1	1.1	3.5	3.4
11	12	1	3.4	1.4	2.2	4
12	13.7	1	2.3	1	2.4	7

[c] Stim RFI score: the sum of CD40, CD40L, CD80, OX40, and OX40L

Mean RFI score for immunostimulatory proteins: **16.2**

Mean Stim/Supp ratio for non-MTEX: **1.4**

STable 2c. Results of on-bead flow cytometry analysis of proteins carried by exosomes from healthy donors

Melanoma associated antigens (MAA)

Healthy donor	MAA RFI score[a]	CSPG4	TRYP2	MelanA	Gp100	VLA4
13	8.2	1	1	1	1	4.2
14	7	1	1	1	3	1
15	5	1	1	1	1	1
16	5.8	1	1	1	1	1.8
17	6	1	2	1	1	1
18	7.8	1	1	1	1	3.8

[a] MAA RFI score: the sum of CSPG4, TRYP2, MelanA, Gp100, and VLA4

Mean RFI score for MAA: **6.6**

Immunosuppressive proteins

Healthy donor	Supp RFI Score[b]	PD1	PDL-1	CD39	CD73	Fas	FasL	LAP-TGFβ	TRAIL	CTLA-4
13	7.1	5.4	1	1.1	1	4.9	1	1	1	1
14	13.5	5.2	1.8	1	1	5.6	2.3	1	2.3	4.1
15	7.8	9.2	1	1.1	1.1	3.8	1	1	1.6	1
16	11.2	6.6	1	1	1	2.9	2.1	2.1	1	3
17	8.8	10	1	1	1	5	1	1	1	2.8
18	9	6	1	1	1	2.7	3	1	1	1

[b] Supp RFI score: the sum of PDL-1, CD39, CD73, FasL, LAP-TGFβ, TRAIL, and CTLA-4

Mean RFI score for immunosuppressive proteins: **9.6**

Immunostimulatory proteins

Healthy donor	Stim RFI score[c]	CD40	CD40L	CD80	OX40	OX40L
13	13.8	1	1	1.6	5.4	4.8
14	20.2	1.3	4.4	3.5	4.4	6.6
15	21	1.2	1	2.9	10.4	5.5
16	19.1	1	1	1	9	7.1
17	24	1	4.1	2.3	7	9.6
18	24	1.2	3	3.1	10	6.7

[c] Stim RFI score: the sum of CD40, CD40L, CD80, OX40, and OX40L

Mean RFI score for immunostimulatory proteins: **20.4**

Mean Stim/Supp ratio for healthy donor exosomes**: 2.2**
